# Protective effects of *Cordyceps militaris* against hepatocyte apoptosis and liver fibrosis induced by high palmitic acid diet

**DOI:** 10.3389/fphar.2024.1438997

**Published:** 2025-01-09

**Authors:** Wan-Ting Tsai, Jiro Hasegawa Situmorang, Wei-Wen Kuo, Chia-Hua Kuo, Shinn-Zong Lin, Chih-Yang Huang, Tsung-Jung Ho

**Affiliations:** ^1^ Office of Superintendent, Hualien Tzu Chi Hospital, Buddhist Tzu Chi Medical Foundation, Hualien, Taiwan; ^2^ Institute of Medical Sciences, Tzu Chi University, Hualien, Taiwan; ^3^ Center for Biomedical Research, National Research and Innovation Agency (BRIN), Cibinong, Indonesia; ^4^ Department of Biological Science and Technology, College of Life Sciences, China Medical University, Taichung, Taiwan; ^5^ Ph.D. Program for Biotechnology Industry, China Medical University, Taichung, Taiwan; ^6^ School of Pharmacy, China Medical University, Taichung, Taiwan; ^7^ Department of Sports Sciences, University of Taipei, Taipei, Taiwan; ^8^ Bioinnovation Center, Buddhist Tzu Chi Medical Foundation, Hualien, Taiwan; ^9^ Department of Neurosurgery, Hualien Tzu Chi Hospital, Buddhist Tzu Chi Medical Foundation, Hualien, Taiwan; ^10^ Cardiovascular and Mitochondrial Related Disease Research Center, Hualien Tzu Chi Hospital, Buddhist Tzu Chi Medical Foundation, Hualien, Taiwan; ^11^ Graduate Institute of Biomedical Sciences, China Medical University, Taichung, Taiwan; ^12^ Department of Medical Research, China Medical University Hospital, China Medical University, Taichung, Taiwan; ^13^ Department of Biotechnology, Asia University, Taichung, Taiwan; ^14^ Center of General Education, Buddhist Tzu Chi Medical Foundation, Tzu Chi University of Science and Technology, Hualien, Taiwan; ^15^ Integration Center of Traditional Chinese and Modern Medicine, Hualien Tzu Chi Hospital, Buddhist Tzu Chi Medical Foundation, Hualien, Taiwan; ^16^ Department of Chinese Medicine, Hualien Tzu Chi Hospital, Buddhist Tzu Chi Medical Foundation, Hualien, Taiwan; ^17^ School of Post-Baccalaureate Chinese Medicine, College of Medicine, Tzu Chi University, Hualien, Taiwan

**Keywords:** fatty liver disease, *Cordyceps militaris*, liver fibrosis, palmitic acid, hepatocyte apoptosis, mitochondrial dysfunction

## Abstract

**Background:**

Fatty Liver Disease (FLD) progresses from steatosis to steatohepatitis and, if left untreated, can lead to irreversible conditions such as cirrhosis and hepatocarcinoma. The etiology of FLD remains unclear, but factors such as overconsumption, poor diet, obesity, and diabetes contribute to its development. Palmitic acid (PA) plays a significant role in FLD progression by inducing apoptosis, inflammation, oxidative stress, and endoplasmic reticulum (ER) stress in hepatocytes. *Cordyceps militaris* (CM), a fungus with various biological activities, including antioxidant properties is examined both *in vitro* and *in vivo* to assess its effectiveness in mitigating PA-induced hepatocyte apoptosis and preventing FLD progression.

**Purpose:**

This study aims to investigate the potential and mechanism of CM in combating FLD, particularly in inhibiting hepatocyte apoptosis.

**Methods:**

*In vitro* studies utilized Clone9 hepatocytes treated with PA to simulate FLD conditions. The effects of CM ethyl acetate extract (EAECM) on apoptosis, mitochondrial function, ER stress, inflammation, and oxidative stress were evaluated. *In vivo* experiments involved FVB mice fed a NASH diet containing high levels of PA to induce FLD, with powdered CM administered orally to assess its impact on body weight, fasting blood glucose level, liver health, fibrosis, and markers of ER stress, inflammation, and oxidative stress.

**Results:**

EAECM demonstrated protective effects against PA-induced apoptosis, mitochondrial dysfunction, ER stress, inflammation, and oxidative stress *in vitro*. *In vivo*, powdered CM supplementation attenuated body weight gain, improved fasting blood glucose level, prevented hepatomegaly, reduced serum triglycerides, and inhibited liver fibrosis. Furthermore, powdered CM treatment mitigated ER stress, inflammation, and oxidative stress in the liver of mice receiving a NASH diet.

**Conclusion:**

*C. militaris* holds promise as a therapeutic agent for FLD, as evidenced by its ability to alleviate PA-induced hepatocytes damage and hinder FLD progression in mice. Further research is warranted to identify the active compounds responsible for its beneficial effects and to explore its potential clinical applications in treating FLD.

## 1 Introduction

Fatty Liver Disease (FLD) is a progressive condition, and at its latest stage, such as cirrhosis, it becomes irreversible and eventually leads to hepatocarcinoma (Clarke et al., 2014; Bugianesi, 2007). In the initial stage, FLD is characterized by a relatively simple accumulation of lipids in the liver known as steatosis. If this phenomenon persists, the disease progresses to a more advanced stage known as steatohepatitis, marked by increased inflammation, cell damage, and elevated liver enzymes (Vernon et al., 2011). Typically, symptoms such as jaundice, itchy skin, and fatigue become apparent at this stage. The etiology of fatty liver remains elusive, although factors such as overconsumption, poor diet, obesity, and diabetes pose a risk for developing this disease. Research has shown that a high level of palmitic acid (PA) is a key contributor and predictor in the development of FLD (Cansancao et al., 2018; Jiang and Sun, 2022). One of the key mechanisms, apart from its contribution to increased lipid accumulation, is its ability to induce cell death in hepatocytes, even in the early stages of FLD. Since PA content is high in meat, such as pork, beef, butter, and plant-based oils, high consumption of these foods might increase the incidence and exacerbate the progression of FLD (Perdomo et al., 2019). *In vitro* and *in vivo* studies have shown that PA’s principal mechanism for inducing cell death is by increasing mitochondrial fragmentation, endoplasmic reticulum (ER) stress, leading to an increase in mitochondrial reactive oxygen species (ROS), apoptosis, and disturbances in protein synthesis and lipid metabolism (Zhang et al., 2012; Perry et al., 2018; Eynaudi et al., 2021). Currently, therapies to combat FLD are limited, with most focusing on lifestyle modifications. While this is important, no single drug has been specifically approved for FLD. However, drugs used for diabetes or dyslipidemia, such as PPARγ agonists and statins, are often employed in FLD treatment (Athyros et al., 2010; Lange et al., 2022).

In this study, we investigated the prospect of *C. militaris* (CM) to combat FLD in both *in vitro* and *in vivo* settings. In the *in vitro* study, we found that the ethyl acetate extract (EAE), but not the water extract of CM, concentration-dependently prevents apoptosis induced by PA. CM is a fungus that grows as a parasite in insects or their larvae as hosts. It is widely used in Chinese medicine, and research has shown its broad range of beneficial biological activities, such as powerful antioxidant properties, anti-aging effects, anti-inflammation, blood glucose reduction, restoration of lipid imbalance, and even increased fertility (Shweta et al., 2023). Some of the known active compounds in CM are cordycepin, ergosterol, and polysaccharides.

While previous studies have also demonstrated the beneficial effects of CM in water extract or Cordycepin, a main constituent of CM, in combating FLD (Gong et al., 2021; Choi et al., 2014), the aspect of apoptosis, a key factor in the etiology of FLD, in the mechanism of CM has not been studied. Our study addresses this gap by demonstrating that the ethyl acetate extract (EAE) of CM, but not the water extract, prevents PA-induced apoptosis in hepatocyte culture in a concentration-dependent manner. Furthermore, treatment with powdered fruiting bodies of CM also shows protection from a high-PA diet in the *in vivo* model, attenuating the progression of FLD.

## 2 Materials and methods

### 2.1 Materials

Clone9 cell was purchased from ATCC (Manassas, VA, United States). Dulbecco’s Modified Eagle’s Medium (DMEM), phosphate buffered saline (PBS), and 100X penicillin-streptomycin (10,000 U/mL) were purchased from Life Technologies Corporation (Grand Island, NY, United States). Fetal bovine serum (FBS) was purchased from Hyclone (Logan, UT, United States). BioTrace™ nitrocellulose transfer membrane was purchased from Pall (Port Washington, NY, United States). Eosin (318,906) reagent and Glucose (G7021) purchased from Sigma Aldrich (St. Louis, MO, United States). Dichlorodihydrofluorescein diacetate (DCFDA) (601,520) was purchased from Cayman (Ann Arbor, MI, United States). CCK-8 cell counting kit was purchased from Vazyme (Nanjing, China). Antibodies against ATF4 (#11815), cleaved-caspase 3 (#9664), p-Drp ser616 (#3455), CHOP (#2895), eIF2α (#9722), p-eIF2α (#9721), p-PERK (#3179) p-P65 (#3033L) and COX2 (#12282) were purchased from Cell Signaling Technology (Danvers, MA, United States). Antibody against p-Drp ser637 (GTX50911) was purchased from Genetex (Irvin, CA, United States). Mason trichrome stain kit (ab150686), antibodies against P65 (ab32536) and total OXPHOS (ab110413) were purchased from Abcam (Cambridge, United Kingdom). Antibodies against Nrf2 (A1244), and p-Nrf2 (AP1133) were purchased from Abclonal Technology (Woburn, MA, United States). Antibodies against catalase (sc-271803), cytochrome c (sc-13560), Drp (sc-271583), GAPDH (sc-32233), IL6 (sc-32296), SOD2 (sc-133134), TNFα (sc-52746), mouse and rabbit secondary antibodies were purchased from Santa Cruz Biotechnology (Dallas, TX, United States). Rabbit green (A11008), Rabbit red (A11012) secondary antibodies for immunofluorescence, and DAPI (R37606) were purchased from Invitrogen (Waltham, MA, United States). Immobilon Western Chemiluminescent HRP Substrate was purchased from Millipore (Burlington, MA, United States). Immunohistochemistry staining kit (PK-8200), and hematoxylin (H3401) reagent was purchased from Vector Laboratories (Newark, CA, United States). NASH diet was purchased from Research Diets (D17010103i, NJ, United States). Glucose strips (ACCU-CHEK) were purchased from Roche (Indianapolis, IN, United States). Strips for TG, total cholesterol, AST, and ALT measurement were purchased from Arkray (Japan).

### 2.2 Preparation of ethyl acetate or water crude extract of *C.militaris*

*C. militaris* was sourced from the Department of Chinese Medicine at Tzu Chi University, Hualien, Taiwan. The identification was carried out by Dr. Tamilselvi Shanmugam from China Medical University Hospital, Taiwan, and Prof. Tsung-Jung Ho from Department of Chinese Medicine at Tzu Chi University, Taiwan. The fungus was identified through morphological examination and confirmed by referencing the Taiwan Herbal Pharmacopoeia 2018 (Version III, Chinese edition). For the ethyl acetate extract preparation, 15 g of powdered fruiting bodies of *C. militaris* (CM) were weighed and subsequently diluted up to 50 mL with ethyl acetate, then shaken for 48 h. For the water extract, after being diluted up to 50 mL, the solution was subjected to boiling for 1 h. The resulting solutions were subsequently subjected to centrifugation at 1,000 × g for 1 h at a temperature of 4°C. The resulting supernatants were further processed by filtration through a 0.22 µm membrane filter and subsequently freeze-dried until complete solvent evaporation.

### 2.3 LC–MS/MS analysis

Three batches of EAECM samples were sent to a biotechnology company (PRO TECH) for HPLC and MS analysis. A total of 100 μL EAECM and 400 μL acetonitrile were vortexed to mix, incubated at −20°C for 30 min, and centrifuged at 15,000 g for 10 min. All analyses were performed on the Agilent 1,260 HPLC system with Phenomenex Luna HILIC-200 A column (50 mm × 2.0 mm i. d., 3.0 um) and AB Sciex Instruments QTRAP 5500.

### 2.4 Culture of Clone9 hepatocyte

Clone9 culture was similar with previous study (Loh et al., 2023). Clone9 cells were maintained in DMEM supplemented with 10% FBS and 1X penicillin-streptomycin at 37°C in a humidified 5% CO2 incubator. Once the cells reached around 90%–100% confluence, they were seeded in 6-well or 12-well culture plates for experiments. After reaching around 70% confluence, the growth media was replaced with serum free DMEM to make the cells quiescent for overnight.

### 2.5 Viability assay

Clone9 cells were cultured in 12-well plates, after they reached 70% confluence, the growth media was replaced with DMEM without serum and the cells were incubated at 37°C for overnight before further treatment. After treatment, the viability of the cells was measured by using CCK-8 assay kit. Briefly, CCK-8 solution was mixed with the growth media (20 μL/mL) and 500 μL was added to each well and incubated for 2 h at 37°C. The absorbance at 460 nm was measured with Epoch™ BioTek Instruments microplate spectrophotometer.

### 2.6 Cell apoptosis analysis

After treatment, the cells were trypsinized, centrifuged, washed with PBS, and stained with FITC Annexin V Apoptosis Detection Kit in binding buffer for 15–30 min, and detected by BD FACSLyric™ Clinical Flow Cytometry System within 1 h (San Jose, CA, United States). The statistical analyses were based on the 10,000 cells per event.

### 2.7 Mitochondrial ROS detection

The MitoSOX molecular probe was used to detect mitochondrial ROS generation in Clone 9 cells. After treatment, the media was removed, and the cells were washed with PBS one time. Then, the MitoSOX (1 μM) solution was added, and the cells were incubated for 20 min in a 37°C incubator. After incubation, the MitoSOX solution was removed, and the cells were subjected to fluorescence imaging or flow cytometry analysis with the Ex/Em wavelength in the range of 510/580 nm.

### 2.8 Analysis of mitochondrial membrane potential

Analysis of mitochondrial membrane potential was similar with previous study (Taufani et al., 2023). 5′,6,6′-tetrachloro-1,1′,3,3′-tetraethylbenzimidazolylcarbocyanine iodide (JC-1) dye was used to detect mitochondrial membrane potential. First, the JC-1 working solution was prepared by mixing ×200 of JC-1 stock solution (1 mg/mL), dd water, and growth media to get a 2.5 μg/mL final concentration. Next, the working solution was added to the cells, followed by incubation for 30 min at 37°C. The stained cells were then subjected to fluorescence imaging or flow cytometry analysis with the Ex/Em wavelength in 520/590 nm and 490/530 nm for JC-1 aggregates and JC-1 monomer, respectively.

### 2.9 ROS detection

ROS detection protocol was similar with previous study (Situmorang et al., 2023). The level of intracellular ROS was determined using the DCFDA assay and analyzed by flow cytometry. The protocol involved washing the cells three times with PBS, adding DCFDA (1.5 μM in PBS), and incubating for 30 min. Following the incubation, the cells were washed once with PBS, trypsinized, and analyzed using flow cytometry.

### 2.10 Western blot analysis

Following treatment, the cells were harvested. The media was removed and the cells were washed with PBS. The mixture of 2X Laemmli buffer and 2X RIPA buffer in 1:1 ratio was added to collect the cells. The total protein was measured using Bradford assay kit. Around 20–30 μg of total protein were separated in 10% SDS-PAGE gel for 100 min and 90 V conditions, and then transferred to a nitrocellulose membrane. The membrane was cut according to the desired protein size, and then incubated in primary antibody (1:1,000) diluted in 5% BSA at 4°C for overnight. The following day, the primary antibody solution was removed, and the membrane was washed thrice for 5 min each with tris buffered saline containing 1% Tween 20. Thereafter, the membrane was incubated with appropriate secondary antibody (1:5,000) diluted in 5% BSA at RT for 1 h. Finally, chemiluminescent substrate was used to visualize the proteins. The signal was captured by using UVP ChemStudio Plus touch (Analytik Jena; Jena, Germany). The images were quantified using ImageJ (NIH).

### 2.11 Immunofluorescence staining and TUNEL assay analysis

The cells were fixed with 4% paraformaldehyde after treatment for 5 min, followed by washing with PBS thrice for few seconds. Next, 1% BSA was added to block the unspecific binding of antibodies for 30-min at RT. After blocking, the cells were incubated with primary antibody overnight at 4°C. The following day, the primary antibody solution was removed, and the cells were washed with PBS thrice. Finally, a secondary antibody was added for 1 h at RT. The cells were mounted with DAPI solution and covered with coverslips. The images were taken using fluorescence microscopy (Olympus). For the TUNEL assay, after blocking the cell was permeabilised with 0.5% Tween-20 for 10 min. The cells was then incubated in TUNEL reaction buffer at 37°C for 1 h and in DAPI solution for 1 min.

### 2.12 Animal experiment

Animal experiments were carried out in strict accordance with the National Institutes of Health Guide for Care and Use of Laboratory Animals and with the approval of the Institutional Animal Care and Use Committee (IACUC approval no. 112–05) of Hualien Tzu Chi Hospital. Furthermore, all procedures conducted in the animal study were in accordance with the ARRIVE guidelines.

To ensure the highest standards of animal welfare, our animal protocols were reviewed and revised based on feedback from the IACUC committee. This committee, comprising veterinarians and researchers, rigorously evaluated our methods to ensure they minimized animal distress and suffering.

Upon acquiring the animals, they were provided with a 1-week period to acclimatize to the room and cage conditions. Five-week-old male FVB fatty liver prone mice were utilized for this investigation. All mice were housed in a controlled environment (temperature: 23°C ± 1°C; humidity: 55%–60%) under a 12/12-h light/dark cycle, with unrestricted access to food and water.

Intragastric administration of powdered fruiting bodies of CM prepared in 0.5% of carboxy methyl cellulose was carried out in two rat groups at doses of 25 mg/kg and 150 mg/kg (Choi et al., 2014), 5 days a week, over a 20-week period, beginning in the 4th week of the NASH diet. Each group received a volume of 5 mL/kg. The control group only received drinking water (5 mL/kg) as a vehicle for *C. militaris*. No difference in drinking intake was observed throughout the experiment.

### 2.13 Glucose tolerance and blood glucose measurement

Fasting glucose and glucose tolerance experiments were conducted 1 week before animal sacrifice. The mice were fasted for 6 h before the first blood glucose measurement. Subsequently, 1.5 g/kg of glucose in a 20 mL/kg sodium chloride solution was given through intraperitoneal injection. Blood glucose levels were then assessed using a glucose strip test every 30 min up to 120 min, with blood samples obtained from the tip of the mice’s tails.

### 3.14 Serum parameters

The serum blood parameters were assessed by automated analyzer for clinical chemistry (SPOTCHEM EZ SP-4430). Briefly, animals were fasted for 6 h and blood was collected during animal sacrifice. The blood was centrifuged, serum was isolated and then kept at −80°C for later analysis of TG, TC, ALT, and AST.

### 2.15 Thiobarbituric acid reactive substances measurement

Liver tissues were subjected to sonication to disrupt the tissue structure. Subsequently, the protein content was quantified. The TBRAS measurements were performed using an equal amount of protein from each sample, following the instructions provided in the TBRAS kit. In brief, 25 μL of the sample was mixed and vortexed with 25 μL of SDS solution. Then, 1 mL of a color reagent, comprising a mixture of thiobarbituric acid diluted with acetic acid and sodium hydroxide, was added, and the mixture was boiled at 100°C for 1 h. Afterward, the sample was promptly placed on ice to halt the reaction for 10 min, followed by centrifugation, and the absorbance was read at 530 nm using an ELISA reader.

### 2.16 Statistical analysis

The data were presented as mean ± S.E.M. and were statistically analyzed and graphed using GraphPad Prism version 7.0.0. All data were assumed to follow a normal distribution. One-way ANOVA and *post hoc* Dunnett multiple comparisons were conducted. A *p*-value of < 0.05 was regarded as statistically significant.

## 3 Results

### 3.1 EAECM protects hepatocytes and from PA-induced apoptosis

**FIGURE 1:**
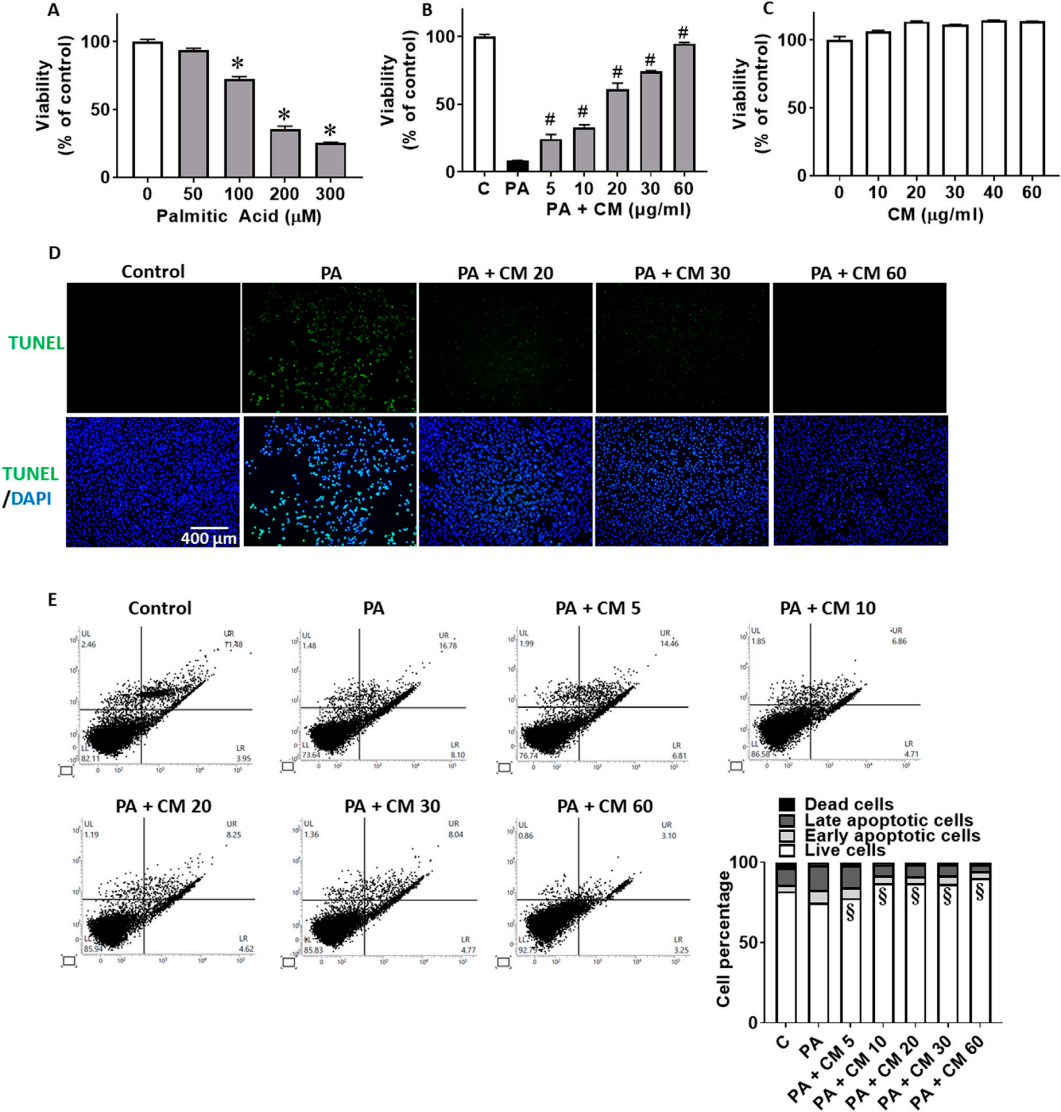
EAECM protects hepatocytes from PA-induced apoptosis. **(A)** Cell viability of Clone9 cells after being challenged with PA for 2 h. **(B)** Cell viability of Clone9 cells after pretreatment with CM for 1 h followed by a challenge with PA (0.3 mM) for 24 h. **(C)** Cell viability of Clone9 cells after treatment with CM for 24 h. **(D)** Representative image of TUNEL assay of Clone9 cells showing the protective effect of CM shown at ×20 magnification. **(E)** PI and Annexin V staining of Clone9 cells showing the protective effect of CM after a challenge with PA for 24 h. (N = 3. *p < 0.05 compared to the control group. ^#^ *p* < 0.05 compared to PA. ^§^ *p* < 0.05 for early and late apoptotic cells compared to PA.

PA has been known to be involved in FLD and it exacerbates the progression of FLD over time (Cansancao et al., 2018). One of the prominent mechanisms of PA is to induce apoptosis in hepatocytes. Using an *in vitro* approach with the Clone9 rat liver cell line, we observed that PA concentration-dependently reduces hepatocyte viability (Figure 1A). EAECM pretreatments for 1 h, in concentrations up to 60 μg/mL, concentration-dependently improved the cell viability of hepatocytes challenged with 300 µM of PA (Figure 1B). While CM alone did not affect the viability of the cells (Figure 1C). We were interested in whether EAECM alone has an effect on viability, and we found that at least up to 60 μg/mL did not affect hepatocyte viability. To further confirm the rescuing effect of EAECM on palmitic acid-induced apoptosis, we followed up the viability results with a TUNEL assay (Figure 1D), PI/Annexin V staining analyzed by flow cytometry (Figure 1E). In those assays, we found similar and consistent effects, indicating that EAECM indeed rescues hepatocytes from PA-induced apoptosis.

### 3.2 EAECM protects hepatocytes from PA-induced mitochondrial dysfunction

**FIGURE 2:**
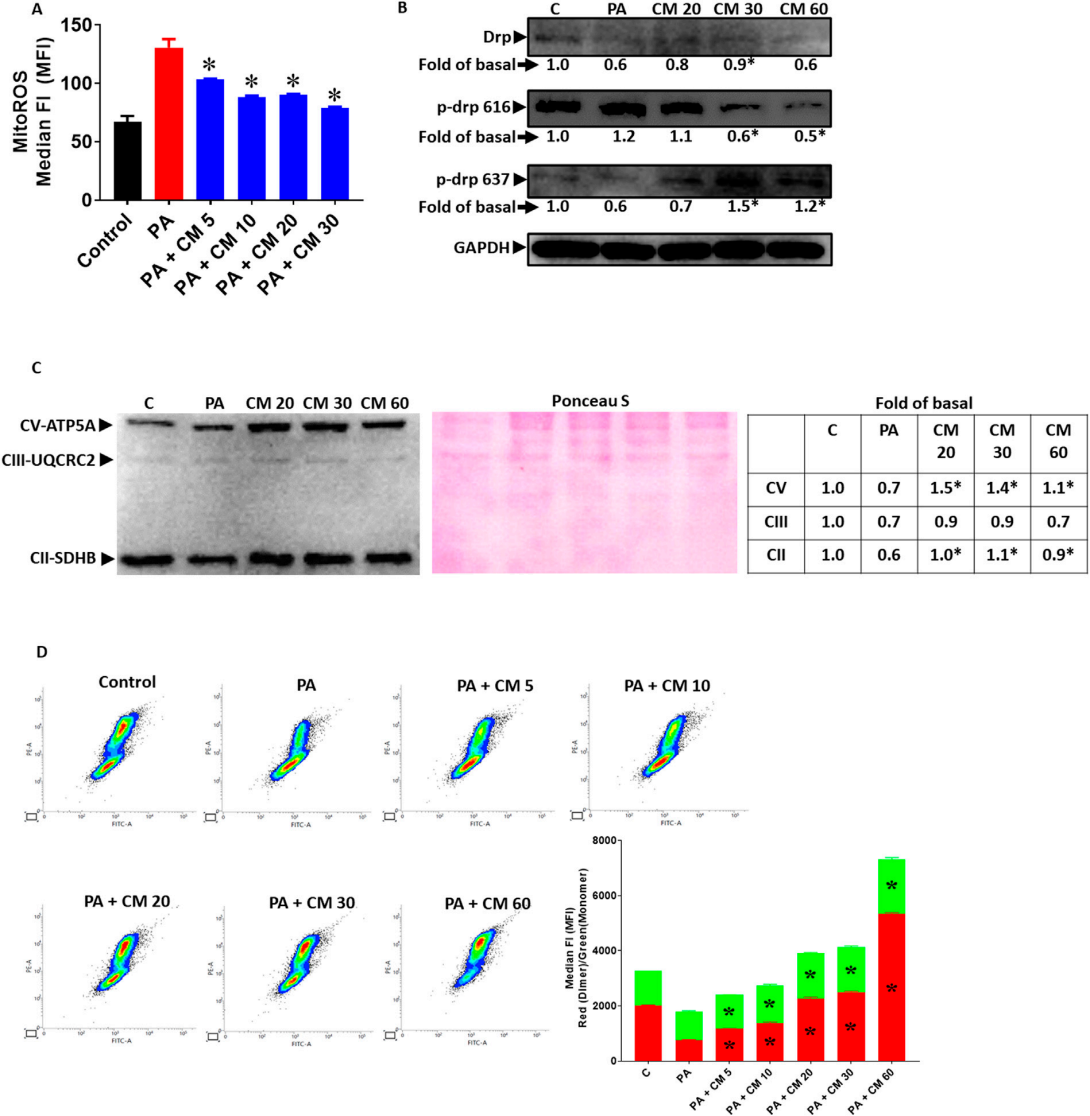
EAECM protects hepatocytes from PA-induced mitochondrial dysfunction **(A)**. Mitochondrial ROS level assessed by flow cytometry, showing the protective effect of CM after a 24-h challenge with PA in Clone9 cells **(B)**. Expression of Drp, p-Drp 616, and p-Drp 637, showing the protection of CM after a 24-h challenge with PA in Clone9 cells. **(C)** Expression of the OXPHOS complex of mitochondria, showing the protection of CM after a 24-h challenge with PA in Clone9 cells. **(D)** Mitochondrial membrane potential assessed by flow cytometry, showing the protection of CM after a 24-h challenge with PA in Clone9 cells. N = 3. *p < 0.05 compared to PA.

In fatty liver condition, the increased level of PA contributes to the progression of fatty liver and causes the liver cells to undergo apoptosis. Mitochondrial damage is one aspect that is involved in apoptosis. One of the key mechanisms by which PA induces apoptosis is by increasing the level of ROS, in which the disturbance of oxidative phosphorylation of the mitochondria is involved (Garcia-Ruiz et al., 2015). In this study, we observed that EAECM alleviate an increase in mitochondrial ROS (Figure 2A) and that EAECM regulated the phosphorylation state of Drp at Ser616 and Ser637, where EAECM reduced the phosphorylation of Drp at Ser616 and increased the phosphorylation of Drp at Ser637, suggesting that EAECM promotes the prevention of mitochondrial fission (Figure 2B) in the hepatocytes treated with PA. We also observed a reduction in the expression of several mitochondrial complexes, including Complex II and V, which EAECM appears to alleviate (Figure 2C). Furthermore, our results also show that PA treatment decreased mitochondrial membrane potential. Interestingly, EAECM treatment concentration-dependently improved the membrane potential even at the highest tested concentration, surpassing the control group (Figure 2D).

### 3.3 EAECM attenuates endoplasmic reticulum stress in hepatocytes induced by PA

**FIGURE 3:**
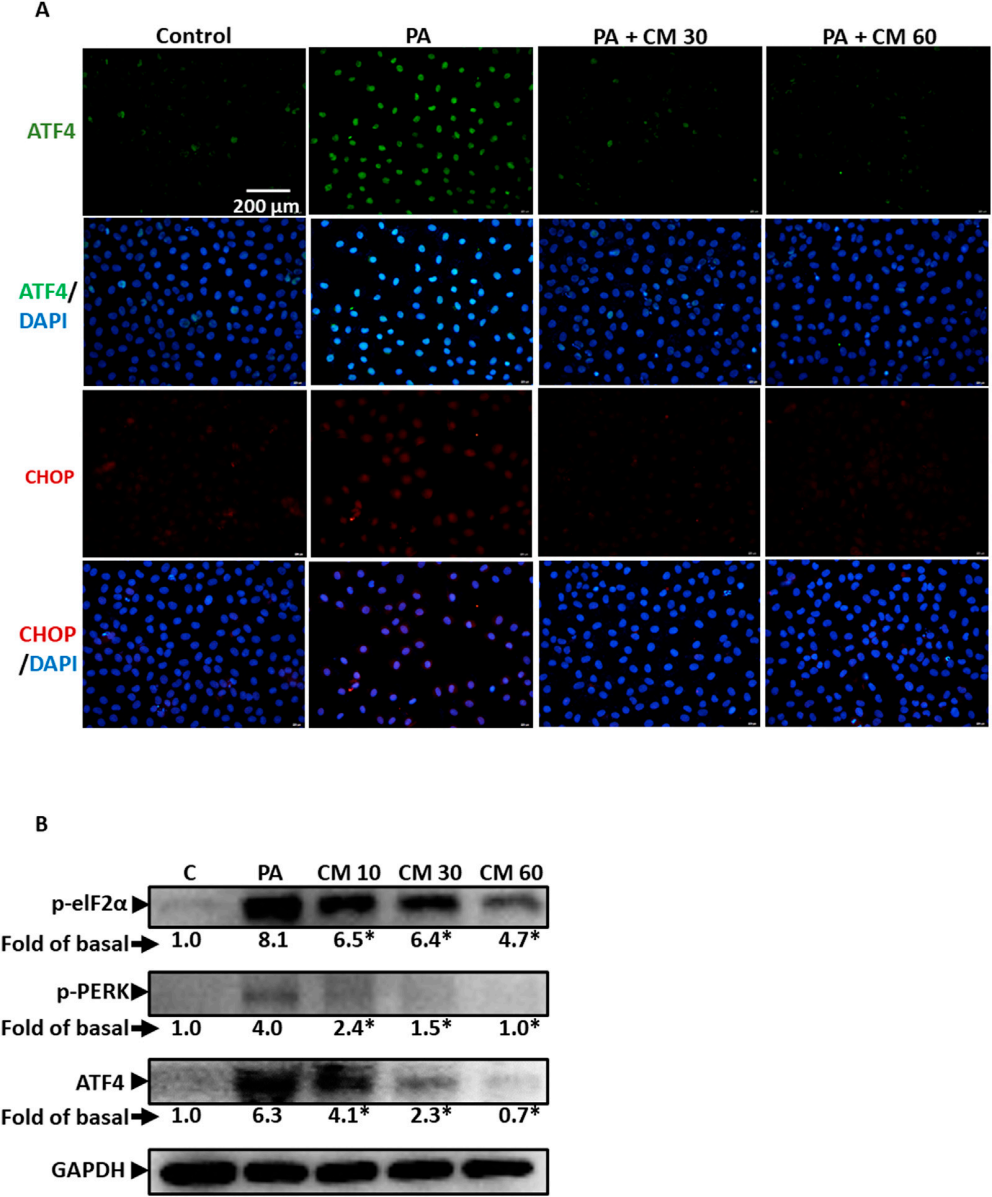
EAECM protects hepatocytes from PA-induced ER stress **(A)**. Immunofluorescence images shown at ×40 magnification of Clone9 cells show ATF4 and CHOP, indicating the protective effect of CM after a 24-h challenge with PA **(B)**. Western blot images demonstrate the expression of ER stress markers such as p-eIF2α, p-PERK, and ATF4, indicating the protective effect of CM after a 24-h challenge with PA in Clone9 cells. N = 3. *p < 0.05 compared to PA.

Endoplasmic reticulum (ER) stress is also involved in fatty liver disease. Research has shown that PA can induce ER stress, although the mechanism is lacking (Zhang et al., 2012). In this study, we showed that PA indeed caused ER stress, indicated by the upregulation of several ER stress markers such as ATF4 and CHOP (Figure 3A), as well as increased phosphorylation of eIF2α and PERK (Figure 3B). Concentration-dependently, we showcased that EAECM reduces the upregulation of these markers, suggesting that EAECM also exerts its protective effects through the regulation of ER function.

### 3.4 Antioxidant enzymes are involved in the protective role of EAECM in attenuating PA-induced oxidative stress

**FIGURE 4:**
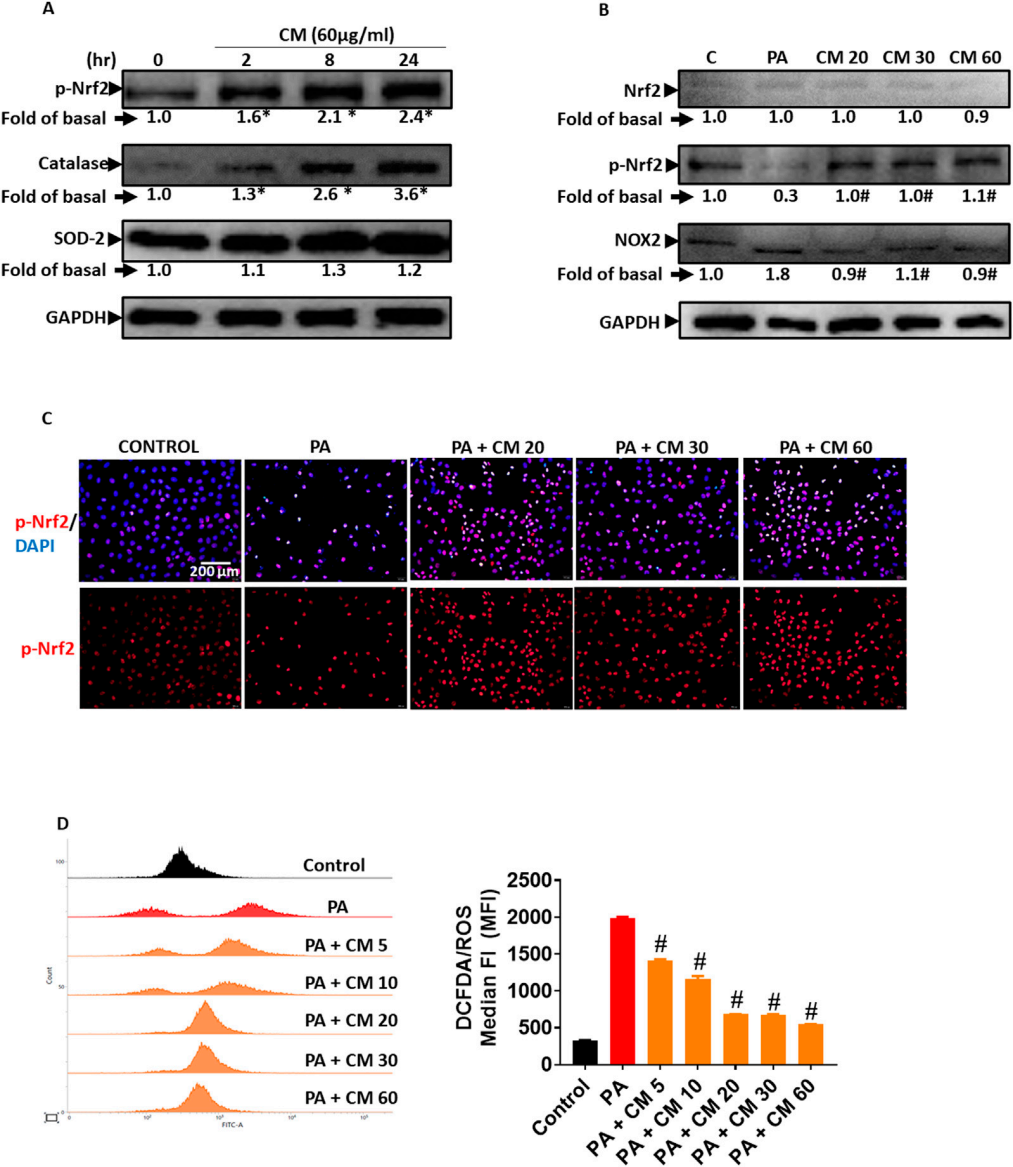
EAECM protects hepatocytes from PA-induced oxidative stress. **(A)** Western blot images demonstrate the expression of antioxidant proteins such as p-Nrf2, catalase (CAT), and SOD2 after a 24-h treatment with CM in Clone9 cells. **(B)** Western blot images demonstrate the expression of antioxidant proteins such as Nrf2, p-Nrf2, and the oxidative marker NOX2, indicating the protective effect of CM after a 24-h challenge with PA in Clone9 cells. **(C)** Immunofluorescence images shown at ×40 magnification of Clone9 cells showing p-Nrf2, indicating the protective effect of CM after a 24-h challenge with PA. **(D)** ROS levels in Clone9 cells, assessed by flow cytometry using DCFDA, indicating the protective effect of CM after a 24-h challenge with PA. N = 3. *p < 0.05 compared to the control group. ^#^ *p* < 0.05 compared to PA.

Ethyl acetate, as a solvent, is usually used to extract phenolic compounds from plants such as flavonoids that possess antioxidant activity. In this study, we evaluated the ability of EAECM to modulate antioxidants in Clone9 cells. Our results revealed that EAECM (60 μg/mL) increased the activity of Nrf2, as indicated by the upregulation of its phosphorylated form at Ser40. Additionally, EAECM (60 μg/mL) also upregulated catalase in a time-dependent manner (Figure 4A). The activity of Nrf2 as an antioxidant regulator was diminished in Clone9 cells treated with PA, accompanied by upregulation of NOX2, which is responsible for superoxide production. On the other hand, EAECM (20, 30, 60 μg/mL) restored the normal levels of NOX2 and phospho-Nrf2 (Figure 4B). In addition, our immunofluorescence results further confirm that under EAECM treatment, the activity of Nrf2, indicated by p-Nrf2, is increased compared to cells treated with PA (Figure 4C). PA has been known to induce oxidative stress, such as ROS, leading to cell death. Treatment with EAECM (5–60 μg/mL) attenuated the increase in ROS, as assessed by DCFDA using flow cytometry (Figure 4D).

### 3.5 EAECM attenuates the inflammation induced by PA

**FIGURE 5:**
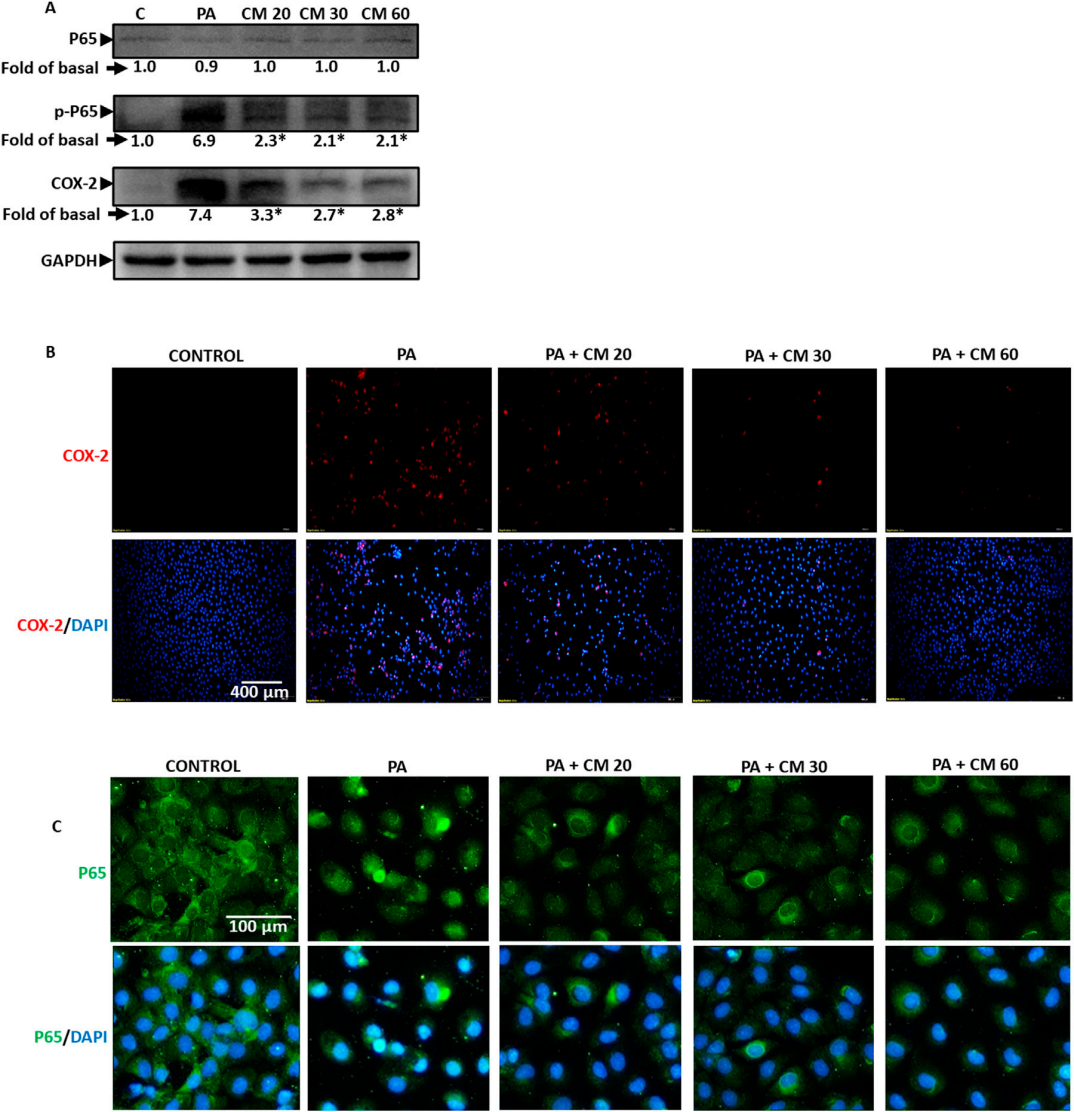
EAECM protects hepatocytes from PA-induced inflammation. **(A)** Western blot images demonstrate the expression of inflammation markers such as P65, p-P65, and COX2, indicating the protective effect of EAECM after a 24-h challenge with PA. **(B)** Immunofluorescence shown at ×20 magnification images of Clone9 cells showing COX2 **(B, C)** P65, indicating the protective effect of EAECM after a 24-h challenge with PA. N = 3. *p < 0.05 compared to PA.

Palmitic acid is known to induce inflammation. In fatty liver disease (FLD), inflammation is one of the triggers that worsen the progress of the disease (Ogawa et al., 2018). Our results revealed that PA increased the phosphorylation of P65, a subunit of NF-kB, and increased the expression of cyclooxygenase 2 (COX2), suggesting an increase in inflammation. Meanwhile, treatment with EAECM (20, 30, 60 μg/mL) attenuated the upregulation of phosphorylation of P65 and cyclooxygenase 2 (COX2) as assessed with Western blot (Figure 5A). Using immunofluorescence, we also showed that treatment with EAECM (20, 30, 60 μg/mL) reduced the expression of COX2 (Figure 5B) and reduced the translocation of P65 into the nucleus (Figure 5C), which strengthens the results of our Western blot.

### 3.6 *Cordyceps militaris* attenuates the increase in body weight and fasting glucose in mice receiving a NASH diet

**FIGURE 6:**
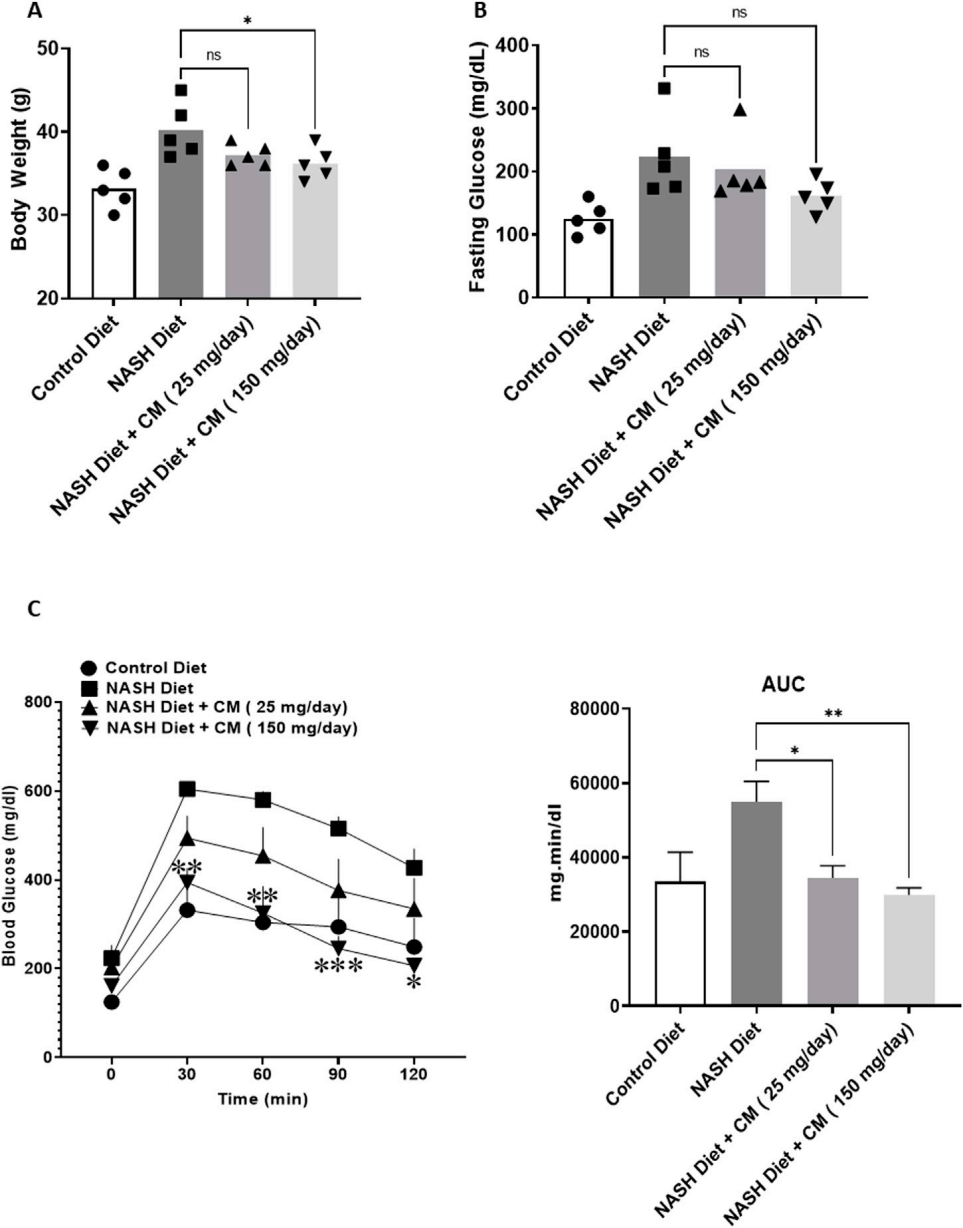
Powdered CM attenuates body weight gain **(A)**, prevents hyperglycemia **(B)**, and improves glucose tolerance **(C)** in FVB mice fed a NASH diet for 20 weeks. N = 5. *p < 0.05, **p < 0.01 compared to NASH diet.

To assess the effect of *C. militaris* (CM) *in vivo*, we employed FVB mice, previously reported to be suitable as an *in vivo* model for fatty liver disease (FLD). A NASH diet was administered over the course of 20 weeks, while powdered fruiting bodies of CM prepared in 0.5% carboxymethyl cellulose (CMC) (25 mg/day and 150 mg/day) were given starting from the 4th week (daily) after the initiation of the NASH diet. At the end of the 20 weeks, we observed that mice given the NASH diet alone had significantly higher body weight compared to mice in the control diet group. Meanwhile, mice given CM showed a trend toward reduction in body weight (Figure 6A). Aside from body weight, fasting blood glucose levels in mice given the NASH diet alone were also significantly higher than those in the control group, while mice treated with CM showed a tendency toward lower fasting blood glucose levels, although this difference was not statistically significant (Figure 6B). We also conducted an additional experiment to assess glucose tolerance in the mice. The results revealed that mice given the NASH diet alone had a higher area under the curve (AUC) than the control group, while mice given CM showed a dose-dependent reduction in the AUC comparable to the control group. In a time-to-time comparison, CM (150 mg/day) showed statistically lower blood glucose levels compared to the NASH diet alone (Figure 6C).

### 3.7 *Cordyceps militaris* prevents hepatomegaly and shows a tendency to reduce serum triglycerides and total cholesterol in mice on a NASH diet

**FIGURE 7:**
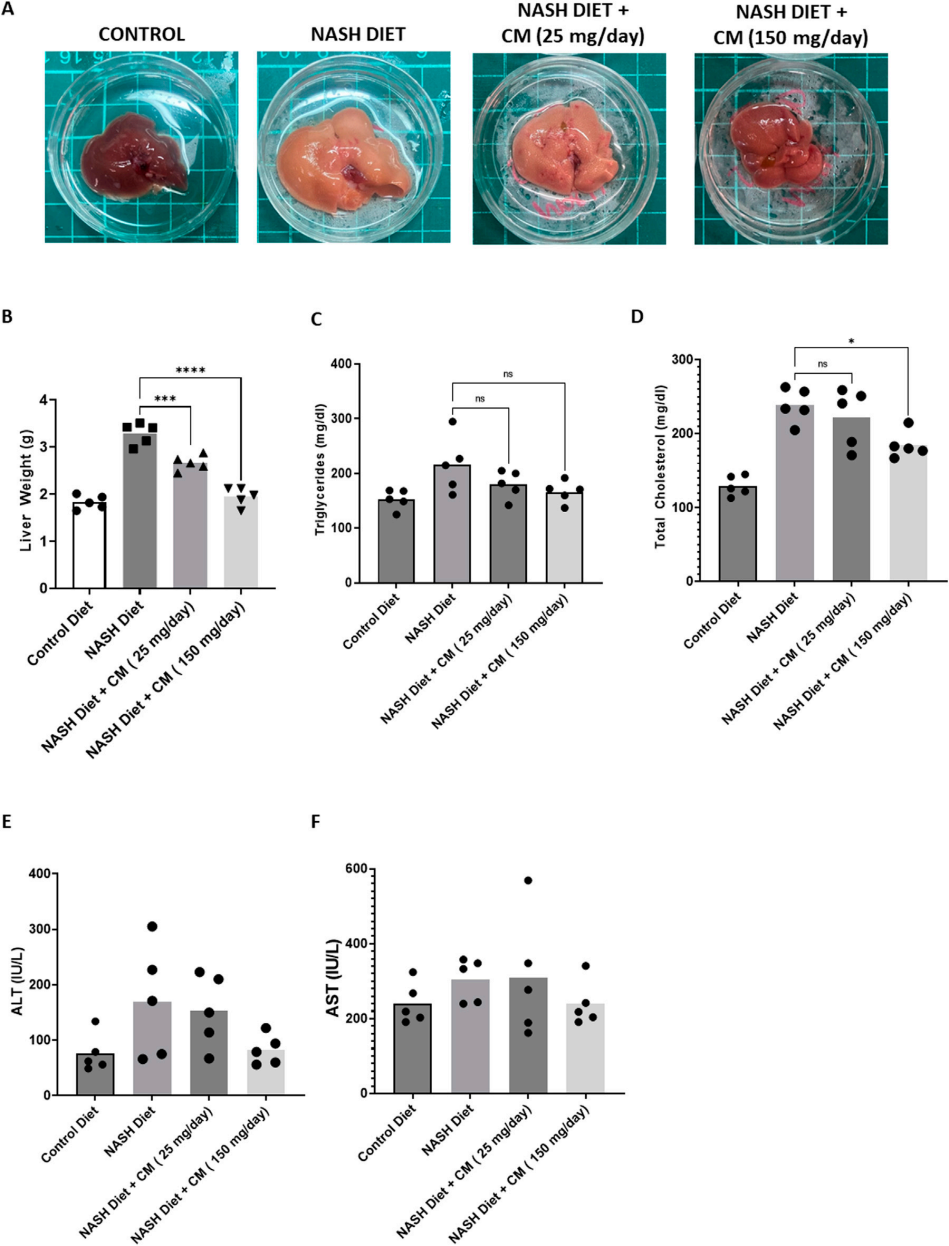
Powdered CM prevents hepatomegaly **(A, B)** and shows a tendency to reduce elevated serum triglycerides and total cholesterol **(C, D)**, although these changes are statistically insignificant. Powdered CM treatment do not affect serum ALT **(E)** and AST **(F)** levels. N = 5. *p < 0.05, ****p < 0.0001 compared to NASH Diet.

One symptom of FLD is hepatomegaly (Basaranoglu and Neuschwander-Tetri, 2006). Our results revealed that mice given a NASH diet for 20 weeks had an increase in their liver size (Figure 7A) and liver weight (Figure 7B). Mice given CM dose-dependently reduced liver size and weight. The results of serum triglycerides also showed that the NASH diet increased serum triglyceride levels (Figure 7C) and total cholesterol (Figure 7D). CM treatment tended to reduce triglyceride levels (statistically insignificant) and significantly reduced total cholesterol levels at a dose of 150 mg/kg. We also observed the liver enzymes ALT (Figure 7E) and AST (Figure 7F); however, the NASH diet or combined with CM treatment did not show any change compared to the control group.

### 3.8 *Cordyceps militaris* prevents liver fibrosis, dysregulated OXPHOS complex, and cell death in mice receiving a NASH diet

**FIGURE 8:**
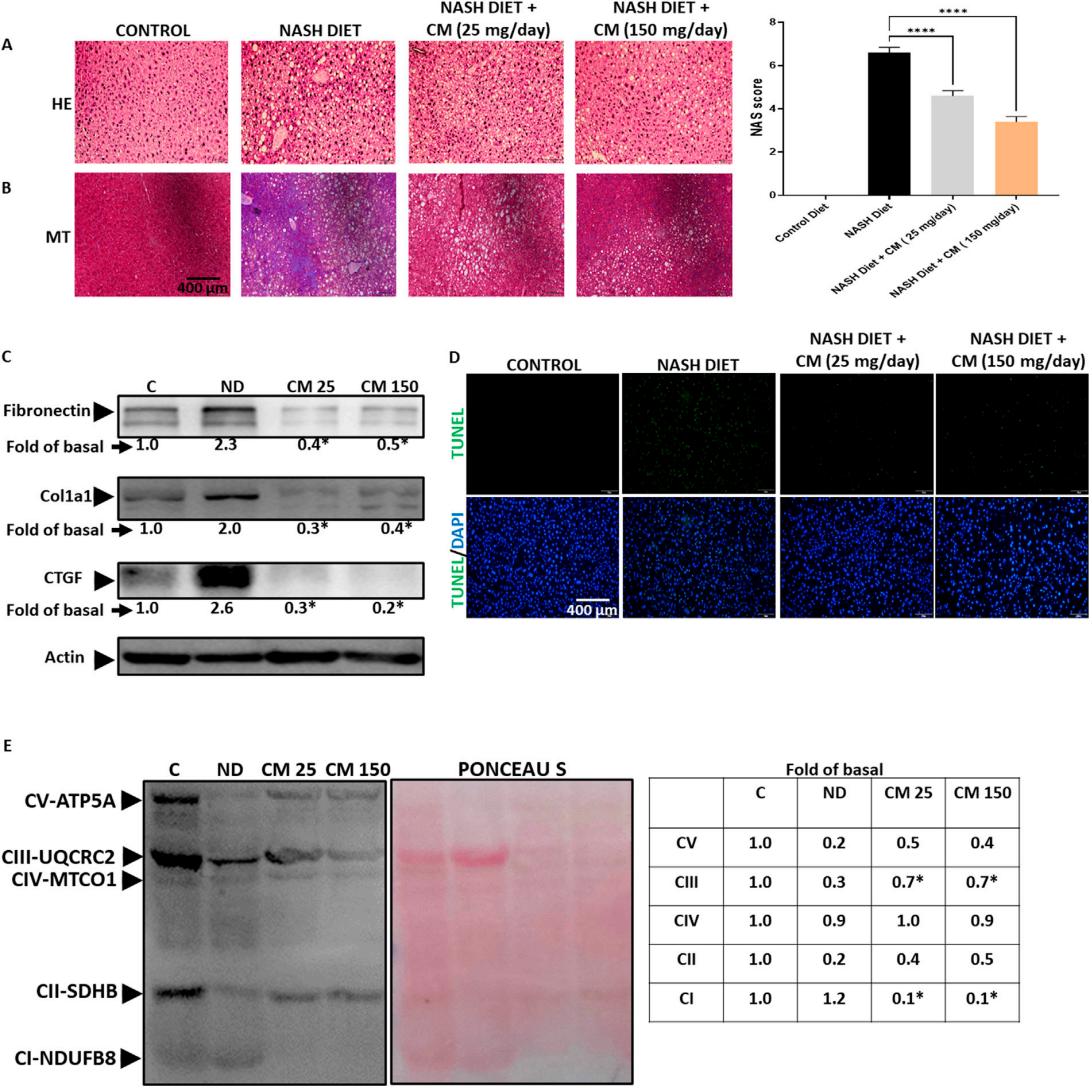
Powdered CM prevents fibrosis, mitochondrial dysfunction, and apoptosis in the FVB mice liver. **(A)** Image shown at ×20 magnification demonstrating lipid accumulation in the mice liver. The NAS score is determined based on the evaluation of 5 images. **(B)** NASH diet treatment induces liver fibrosis, indicated by blue color, while supplementation with powdered CM prevents fibrosis in the liver. **(C)** Western blot images displaying levels of fibronectin, Col1a1, and CTGF. CM treatment prevents the upregulation of these proteins. **(D)** Representative image shown at ×20 magnification of TUNEL assay of liver tissue, showing the protective effect of CM in preventing apoptosis. **(E)** Western blot images illustrating the OXPHOS complex of mitochondria. The NASH diet results in a reduction in the OXPHOS complex, which is prevented by CM treatment. N = 3. *p < 0.05 compared to NASH diet.

Apoptosis and inflammation in FLD can cause tissue scarring and fibrosis (Lu et al., 2023). At steatohepatitis stage, oxidative phosphorylation (OXPHOS) complex of mitochondria has lost efficiency (Simoes et al., 2018). Mice given a NASH diet for 20 weeks displayed fat accumulation (Figure 8A) and high collagen deposition (Figure 8B), suggesting liver fibrosis occurred. Although CM did not affect fat accumulation, CM treatment prevented liver fibrosis in the mice given a NASH diet. We further confirmed the results using Western blot, and our results showed that CM prevented the upregulation of fibrosis markers such as Fibronectin, Col1a1, and CTGF induced by the NASH diet (Figure 8C). Moreover, using the TUNEL assay, we demonstrated that CM treatment prevented cell death induced by the NASH diet (Figure 8D). Our *in vitro* results showed that the effect of EAECM is prominent in the improvement of mitochondrial function, as shown in Section 3.2. In the *in vivo* model, we showcased that CM treatment yielded consistent results, where CM attenuated the dysregulated expression of oxidative OXPHOS complex induced by the NASH diet (Figure 8E).

### 3.9 *Cordyceps militaris* prevents ER stress, inflammation, and oxidative stress in the liver of mice receiving a NASH diet

**FIGURE 9:**
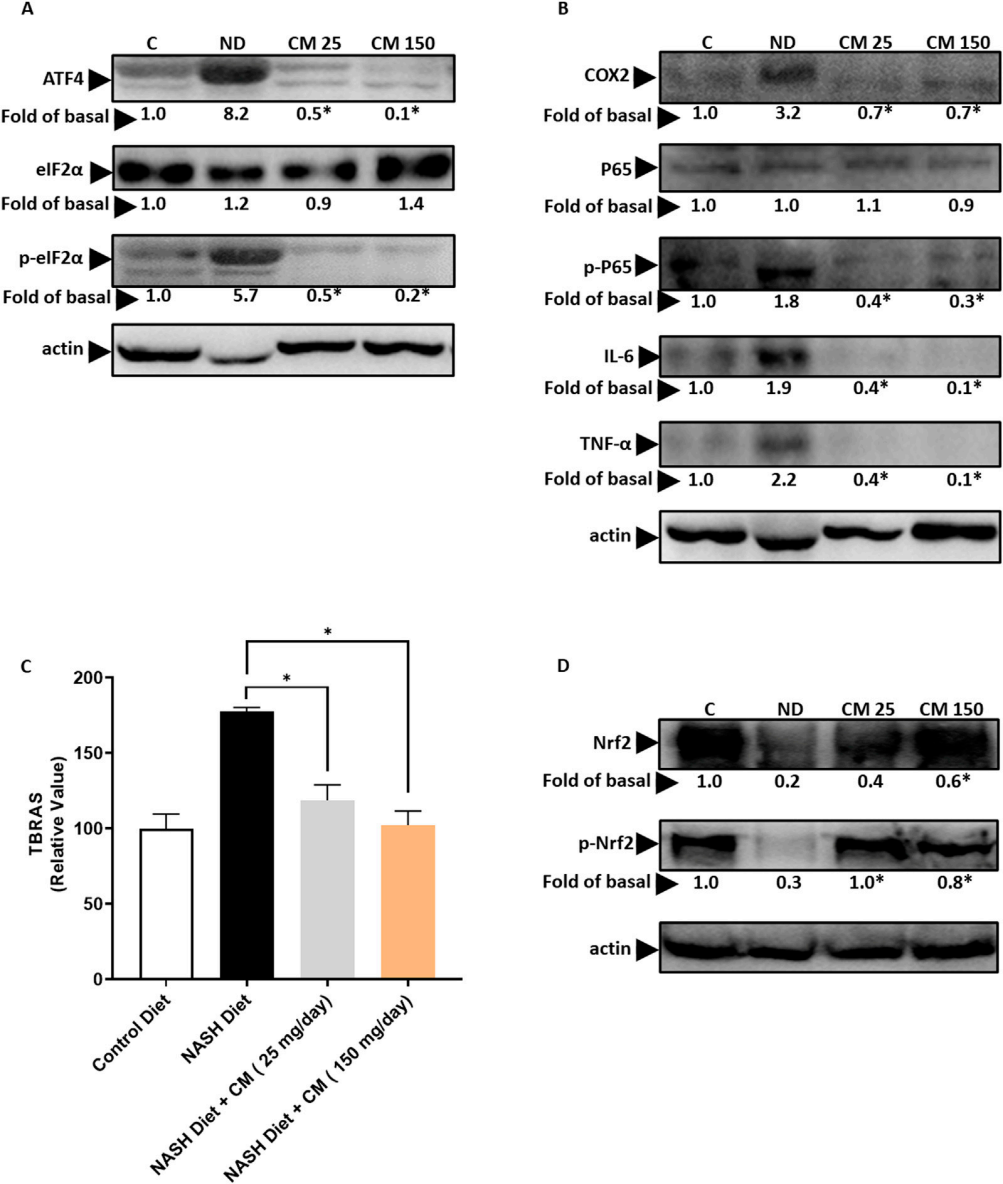
Powdered CM prevents ER stress, inflammation, and oxidative stress in the FVB mice liver. **(A)** Western blot images demonstrate elevated levels of ATF4, eIF2α, and p-eIF2α in the liver of mice treated with a NASH diet. CM treatment prevents their upregulation. **(B)** Western blot images show increased levels of COX2, P65, p-P65, IL-6, and TNF-α in the liver of mice treated with a NASH diet, which are prevented by CM treatment. **(C)** Graph illustrating TBARS levels as an indicator of oxidative stress in the liver. Treatment with powdered CM prevents the increase in TBARS levels. N = 5 *p < 0.05 is compared to ND **(D)** Western blot images display diminished levels of Nrf2 and p-Nrf2 in the liver of mice treated with a NASH diet, which are prevented by CM treatment. N = 3. *p < 0.05 compared to NASH diet.

Our *in vitro* findings demonstrate that EAECM effectively mitigates ER stress, inflammation, and oxidative stress induced by PA. To investigate further, we explored whether CM treatment could offer similar protective effects *in vivo*. As shown in Figure 9A, CM treatment effectively prevented the upregulation of ATF4, and phospho eIF2α, which are indicative of ER stress. In Figure 9B, our results showed that CM inhibited the elevation of inflammation markers such as COX2, phospho P65, TNFα, and IL6 in the mice liver triggered by a NASH diet. Furthermore, CM also hindered the increase in TBRAS levels, an indication of oxidative stress, in the liver (Figure 9C). Additionally, our findings suggested that CM prevented the downregulation of Nrf2 and phospho Nrf2 (Figure 9D).

## 4 Discussion

The exact cause of Fatty Liver Disease (FLD) remains elusive despite its similarities to other metabolic disorders like diabetes and obesity. Resmetirom, a thyroid hormone receptor beta (THR-β) agonist, has recently been approved by the US FDA as the only pharmaceutical therapy for fatty liver disease. Its mechanism of action involves increasing fatty acid oxidation, which helps reduce fat accumulation in the liver (Kingwell, 2024).

Elevated levels of palmitic acid (PA), a fatty acid found in both plant oils and animal meat, are known to accelerate FLD progression. Excessive consumption of PA-rich foods increases the risk of developing FLD due to mechanisms such as apoptosis, inflammation, oxidative stress, and ER stress (Scavo et al., 2023; Park et al., 2019). FLD often progresses slowly and can become fatal and irreversible. Diagnosis typically occurs at severe stages when symptoms manifest, as the disease is asymptomatic in its early stages (Mahale et al., 2018; Armstrong et al., 2014). Our study aimed to investigate whether *C. militaris* (CM) could hinder FLD progression to severe stages. We conducted experiments using both *in vitro* and *in vivo* models. In the *in vitro* model, Clone9 hepatocytes were exposed to PA. We demonstrated that the ethyl acetate extract of CM (EAECM) exhibited potential in rescuing hepatocytes from PA-induced apoptosis, inflammation, ER stress, and oxidative stress. Additionally, EAECM improved mitochondrial membrane potential and function, which are decreased by PA. Water extract of CM has previously been shown to improve glucose and lipid profiles in ob/ob mice fed a high-fat diet (Choi et al., 2014). However, our results showed that the water extract of CM failed to mitigate PA-induced apoptosis (data not shown). Both ethyl acetate and water are known to extract key bioactive compounds in CM, such as cordycepin and adenosine. However, ethyl acetate has been reported to produce a lower yield of extract compared to water, which suggests that ethyl acetate may help reduce impurities in the final extract (Oh et al., 2020). This difference may explain why the EAECM was more effective in this study compared to the water extract. To investigate the efficacy of CM *in vivo*, we administered powdered fruiting bodies of CM to FVB mice fed a NASH diet rich in fat, fructose, and PA. A recent study shows that FVB mice are suitable models for fatty liver disease (Karimkhanloo et al., 2023). Two dosages, 25 mg and 150 mg daily for 5 days a week, were tested, with the amounts calculated based on a previous study (Choi et al., 2014). Mice treated solely with the NASH diet displayed elevated fasting blood glucose levels, impaired glucose tolerance, hepatomegaly, and increased serum triglyceride, and total cholesterol levels. Histological analysis revealed fat accumulation and fibrosis in the livers of these mice, resembling steatohepatitis (Younossi et al., 2011). Furthermore, markers of fibrosis, ER stress, inflammation, and oxidative stress were upregulated in the NASH diet group. Treatment with CM at both dosages halted disease progression, as evidenced by reductions in liver size and weight, fibrosis, improvements in mitochondrial function, and reductions in ER stress and oxidative stress markers. In our *in vitro* study, we did not observe any changes in fibrosis markers after treatment with PA. However, *in vivo*, fibrosis markers such as fibronectin, Col1a1, and CTGF were significantly upregulated in mice fed a NASH diet alone. This was further supported by Masson’s trichrome staining, which showed clear indications of fibrosis, marked by an increase in blue staining. This discrepancy between *in vitro* and *in vivo* findings is somewhat expected, as the *in vitro* model involved only hepatocytes, whereas *in vivo*, all liver resident cells, including hepatocytes and hepatic stellate cells, contribute to the fibrosis process. Hepatic stellate cells are known to play a critical role in liver fibrosis progression (Wan et al., 2024). Our results suggest that hepatic stellate cells may play a critical role in fibrosis progression and could serve as therapeutic targets for CM.

We observed that triglyceride levels in the CM-treated groups were similar to those in the NASH group. However, total cholesterol levels were significantly reduced in the group treated with 150 mg/day of CM, suggesting a potential lipid-lowering effect. This finding warrants further investigation, either by extending the treatment duration or by utilizing active compounds from the ethyl acetate extract. In addition, CM treatment also improved glucose control, with mice exhibiting lower fasting blood glucose levels and improved glucose tolerance, particularly at the highest dosage. The usage of CM has been historically prevalent in Chinese medicine, particularly for cardiovascular, liver, kidney diseases, and diabetes. In this study, we identified adenosine and cordycepin (shown in Supplementary Table S1) as the main bioactive compounds in CM, in line with previous studies (Fan et al., 2006). One of the key mechanisms of adenosine identified in our study is its inhibition of ER stress (Shahrestanaki et al., 2019), which prevents apoptosis of hepatocytes. Our results indicate that CM can alleviate mitochondrial dysfunction. Specifically, we observed a decrease in the expression of several mitochondrial complexes: Complex II, III, and V in the liver of rats fed a NASH diet and in the rat hepatocyte challenged with PA. In contrast, Complex I expression was increased, suggesting a compensatory response to the heightened energy demand (Okoye et al., 2023). Interestingly, CM treatment appeared to mitigate the decrease in Complex II, III, and V, while diminishing the expression of Complex I. This modulation of Complex I may partly explain how CM reduces ROS and TBARS levels, though further studies are necessary to fully elucidate the molecular mechanisms by which CM modulates mitochondrial function. Recent studies indicate the potential of EAECM as an anti-diabetic agent, showing cordycepin, mannitol, and adenosylribose as the potential compounds in EAECM (Bui et al., 2023). Cordycepin has been shown to prevent FLD in mice fed a high-fat diet by inhibiting lipid metabolism and accumulation through an autophagy mechanism (Gong et al., 2021; Li et al., 2019). It also reduces pro-inflammatory cytokines, potentially through mechanisms involving polyadenylation and p65 inhibition (Kim et al., 2006; Ashraf et al., 2019). The limitation of our study is that while we used the ethyl acetate extract of CM *in vitro*, we used powdered fruiting bodies of CM for the animal experiments due to limited extraction yield. Hence, the net effect in the animals is likely also affected by compounds not extracted using ethyl acetate. Another limitation is the lack of a positive control group in our experiments. At the time this study was conducted, there were no approved drugs for fatty liver disease. Although resmetirom, a thyroid hormone receptor β agonist, was approved by the FDA in March 2024, it was unavailable during our study. Additionally, we did not analyze the compounds in the water extracts, making it unclear the amount of bioactive compounds present. Therefore, future studies evaluating the differences in bioactive compounds between the water and ethyl acetate extracts are warranted. Despite this, we observed similar results in the *in vivo* study using powdered mycelia of CM. These findings align with previous studies, suggesting that CM might be a potential candidate for treating FLD.

## 5 Conclusion

Although previous studies have shown the beneficial application of CM in fatty liver (Gong et al., 2021; Choi et al., 2014), our study is the first to demonstrate the effect of EAECM on PA-induced apoptosis, oxidative stress, ER stress, and inflammation. Additionally, this study further demonstrates the anti-FLD effect, particularly the prevention of liver fibrosis, of powdered CM supplementation in animals fed a NASH diet high in PA. In conclusion, our findings indicate that EAECM exhibits a protective effect against PA-induced apoptosis, ER stress, oxidative stress, and inflammation. Moreover, supplementation with powdered CM in mice fed a NASH diet protects against hepatomegaly, hyperglycemia, liver fibrosis, oxidative stress, ER stress, and liver inflammation. These results suggest that CM could serve as a promising therapeutic approach for patients suffering from FLD, or as a dietary supplement for healthy individuals to avoid the occurrence of FLD.

## Data Availability

The raw data supporting the conclusions of this article will be made available by the authors, without undue reservation.

## Abbreviations

ALT, Alanine Transaminase; AST, Aspartate Aminotransferase; ATF4, Activating Transcription Factor 4; AUC, Area Under Curve; CHOP, C/EBP-homologous Protein; CM, *Cordyceps militaris*; COX2, cyclooxygenase 2; CTGF, Connective Tissue Growth Factor; EAE, Ethyl Acetate Exract; ER, Endoplasmic Reticulum; FLD, Fatty Liver Disease; IL6, Interleukin 6; NOX2, NADPH oxidase 2; NASH, Nonalcoholic Steatohepatitis; Nrf2, Nuclear Factor Erythroid 2–related Factor 2; OXPHOS, Oxidative Phosphorylation; PA, Palmitic Acid; ROS, Reactive Oxygen Species; TBRAS, Thiobarbituric Acid Reactive Substances; TNF-α, Tumor Necrosis Factor α; TUNEL, Terminal Deoxynucleotidyl Transferase Dutp Nick End Labeling.
