# Corrigendum: Protective effects of *Cordyceps militaris* against hepatocyte apoptosis and liver fibrosis induced by high palmitic acid diet

**DOI:** 10.3389/fphar.2025.1619149

**Published:** 2025-05-09

**Authors:** Wan-Ting Tsai, Jiro Hasegawa Situmorang, Wei-Wen Kuo, Chia-Hua Kuo, Shinn-Zong Lin, Chih-Yang Huang, Tsung-Jung Ho

**Affiliations:** ^1^ Office of Superintendent, Hualien Tzu Chi Hospital, Buddhist Tzu Chi Medical Foundation, Hualien, Taiwan; ^2^ Institute of Medical Sciences, Tzu Chi University, Hualien, Taiwan; ^3^ Center for Biomedical Research, National Research and Innovation Agency (BRIN), Cibinong, Indonesia; ^4^ Department of Biological Science and Technology, College of Life Sciences, China Medical University, Taichung, Taiwan; ^5^ Ph.D. Program for Biotechnology Industry, China Medical University, Taichung, Taiwan; ^6^ School of Pharmacy, China Medical University, Taichung, Taiwan; ^7^ Department of Sports Sciences, University of Taipei, Taipei, Taiwan; ^8^ Bioinnovation Center, Buddhist Tzu Chi Medical Foundation, Hualien, Taiwan; ^9^ Department of Neurosurgery, Hualien Tzu Chi Hospital, Buddhist Tzu Chi Medical Foundation, Hualien, Taiwan; ^10^ Cardiovascular and Mitochondrial Related Disease Research Center, Hualien Tzu Chi Hospital, Buddhist Tzu Chi Medical Foundation, Hualien, Taiwan; ^11^ Graduate Institute of Biomedical Sciences, China Medical University, Taichung, Taiwan; ^12^ Department of Medical Research, China Medical University Hospital, China Medical University, Taichung, Taiwan; ^13^ Department of Biotechnology, Asia University, Taichung, Taiwan; ^14^ Center of General Education, Buddhist Tzu Chi Medical Foundation, Tzu Chi University of Science and Technology, Hualien, Taiwan; ^15^ Integration Center of Traditional Chinese and Modern Medicine, Hualien Tzu Chi Hospital, Buddhist Tzu Chi Medical Foundation, Hualien, Taiwan; ^16^ Department of Chinese Medicine, Hualien Tzu Chi Hospital, Buddhist Tzu Chi Medical Foundation, Hualien, Taiwan; ^17^ School of Post-Baccalaureate Chinese Medicine, College of Medicine, Tzu Chi University, Hualien, Taiwan

**Keywords:** fatty liver disease, *Cordyceps militaris*, liver fibrosis, palmitic acid, hepatocyte apoptosis, mitochondrial dysfunction

In the published article, there was an error in the **Affiliations** for Tsung-Jung Ho. As well as having affiliations 15, 16, and 17, they should also have affiliation 2.

The authors apologize for this error and state that this does not change the scientific conclusions of the article in any way. The original article has been updated.

