# Supplementary material for: Protective effects of Cordyceps militaris against hepatocyte apoptosis and liver fibrosis induced by high palmitic acid diet
Source: Front Pharmacol. 2025 Jan 9;15:1438997. doi: 10.3389/fphar.2024.1438997 (PMC11755097; doi:10.3389/fphar.2024.1438997)
Supplement: Supplementary file 1 [file DataSheet1.pdf]

Figure S1

Instrument Device :

Instrument Categories	Instrument Model
Pump Model	Agilent 1260 G1312B Binary Pump
AutoSampler	Agilent 1260 G1367E HiP ALS Autosampler
Degasser	Agilent 1260 G1379B Degasser
Column	Phenomenex Kinetex-Phenyl-Hexyl-100A (100 mm x 2.1 mm i.d., 2.6 um)
Mass Spectrometer	AB Sciex Instruments QTRAP 5500
Source type	Turbo V Ion Source

HPLC Method Properties :

Duration : 10 min
Injection volume : 5 ul
Mobile Phase : A: 0.1% (v/v) Formic acid / Water ; B: 0.1% (v/v) Formic acid / Acetonitrile
Step Table :

Step	Total Time(min)	Flow Rate(μl/min)	A (%)	B (%)
0	0.10	100	85.0	15.0
1	2.00	100	85.0	15.0
2	5.00	100	70.0	30.0
3	6.00	100	10.0	90.0
4	7.00	100	10.0	90.0
5	8.00	100	85.0	15.0
6	10.0	100	85.0	15.0

Sample Preparation :

A volume of 100 μL of the sample was taken, followed by the addition of 200 μL of methanol. The mixture was vortexed thoroughly to ensure complete dissolution and subsequently incubated at -20°C for 30 minutes. Centrifugation was performed at 15,000 × g for 10 minutes, and the supernatant was collected and diluted 10× with 50% methanol prior to analysis. (Total dilution factor: 30×)

Mass Spectrometer Information :

Scan Type : MRM (Multiple Reaction Monitor)
Polarity : Positive ion mode
Source temperature : 400 ℃
Data acquisition : Analyst 1.5 software

Parameter Table :

CUR (curtain gas) : 20.00 psi
Collision-activated Dissociation (CAD) : High
Electrospray capillary voltage : 5500.00 V
Nebulizing gas (GS1) : 45.00 psi
Heating gas (GS2) : 40.00 psi

Preparation of Calibrators :

The powdered standard was dissolved in methanol to prepare stock solutions of each target compound. These stock solutions were then used to prepare mixed working solutions with concentrations around μg/mL, which were stored at -20°C for future use. Calibration solutions were freshly prepared and serially diluted with 50% methanol to achieve a concentration range of ng/mL.

Quantitation Method :

As the provided standards are not isotope-labeled, the Multiple Point External Standard method was employed for quantitation.

Selected Transitions and Parameter Settings

Analytes	Q1 mass	Q3 mass	DP (V)	EP (V)	CE (V)	CXP (V)
Adenosine	268.2	85.2<br>136.2	180	10	34<br>24	13<br>10
Cordycepin	252.2	119.2<br>**136.2**	220	10	57<br>**21**	10<br>**12**

Note: Red indicates the ion pairs used for quantitation.

Calibration Curve of Standard

Linear regression **Weighting：1/x**

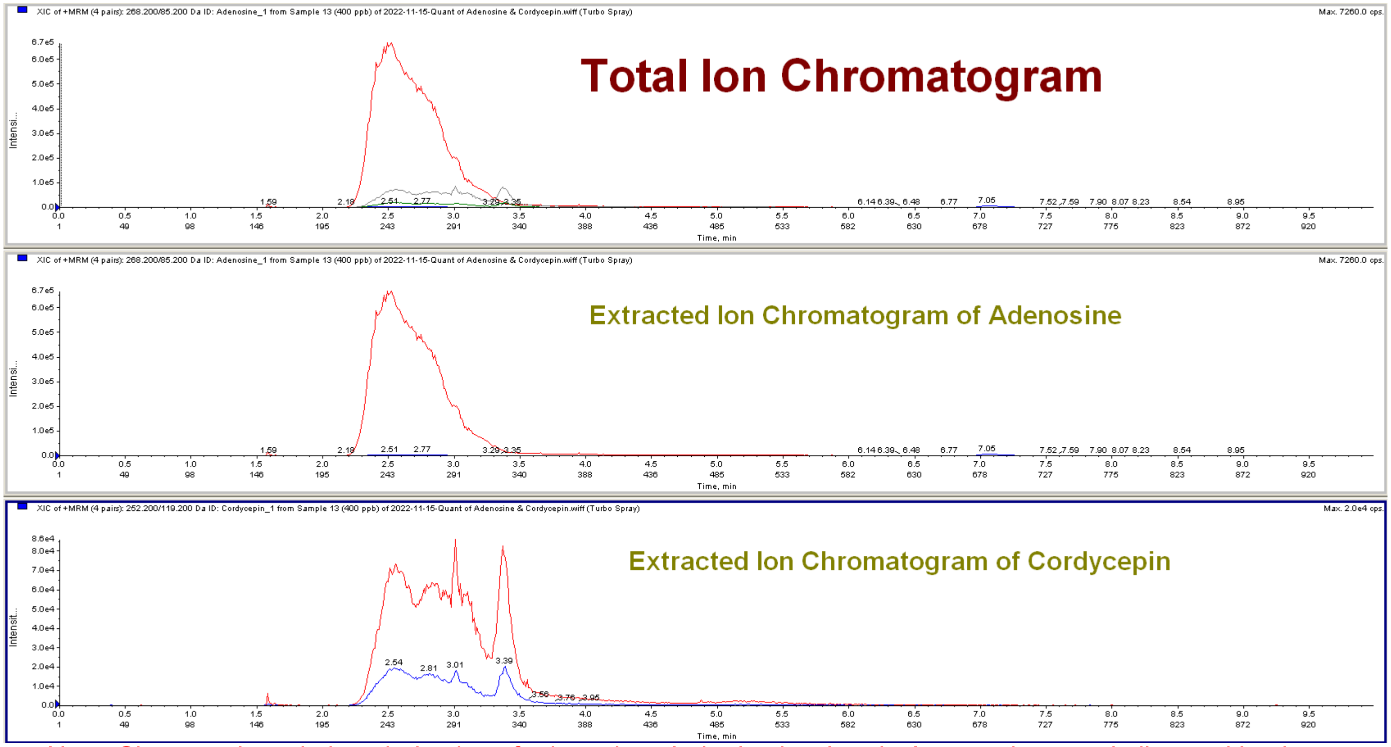

Note: Since cordycepin is a derivative of adenosine, their physicochemical properties are similar, making it difficult to completely separate them under HPLC conditions. However, the EIC chromatogram shows that the selected ion pairs produce distinct signals at their respective retention times, indicating specificity.

Analyte Name **:** Adenosine

Regression Equation : y = 5.56e+004 x + 6.19e+004 (r = 0.9994)

Expected Concentration (ng/ml)	Mean Calculated Concentration (ng/ml)	% Accuracy
0.32	0.27	84.9
1.6	1.69	105.5
3.2	3.29	102.7
8	8.28	103.5
16	16.19	101.2
40	40.36	100.9
80	78.98	98.7
200	210.15	105.1
400	389.90	97.5

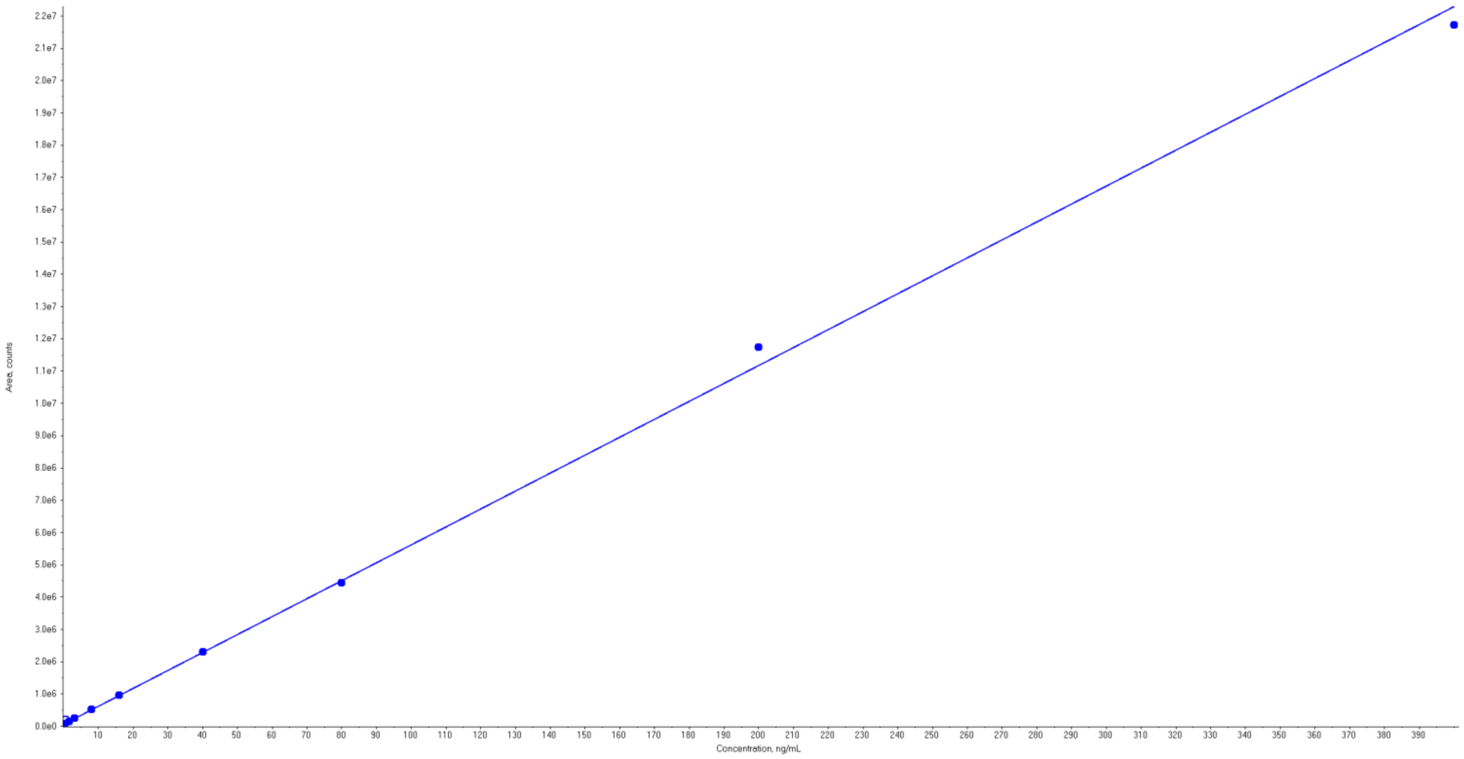

Analyte Name: Cordycepin

Regression Equation: y = 5.68e+003 x + 2.41e+004 (r = 0.9996)

Expected Concentration (ng/ml)	Mean Calculated Concentration (ng/ml)	% Accuracy
0.32	0.26	81.8
0.64	0.67	104.7
1.6	1.72	107.5
3.2	3.29	102.7
8	7.71	96.4
16	16.92	105.7
40	39.27	98.2
80	81.24	101.5
200	207.10	103.5
400	391.58	97.9

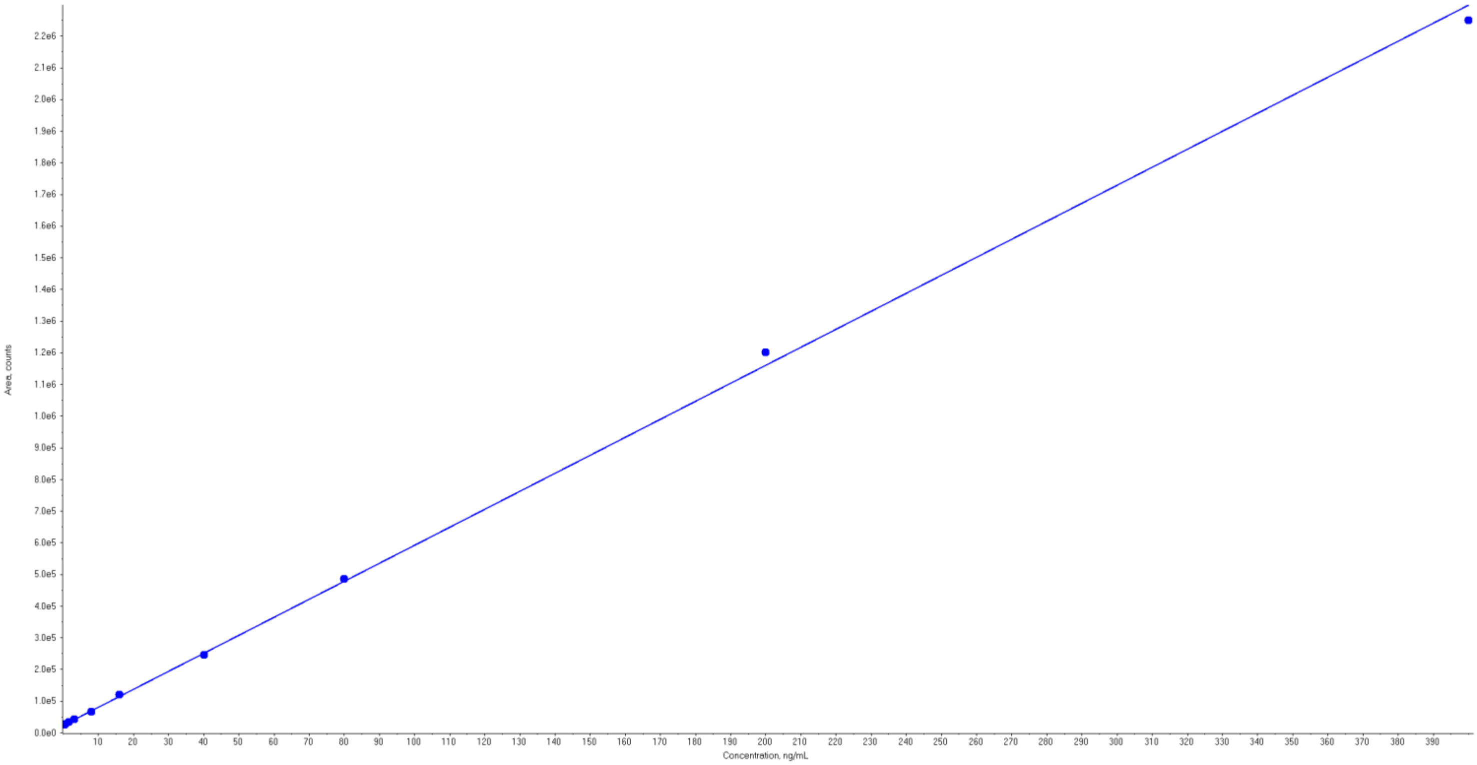

Results

Sample 1

Analyte Name	Spectrum	Calculated concentration (ng/ml)
Adenosine	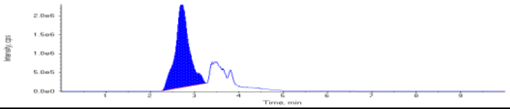	855.00
Cordycepin	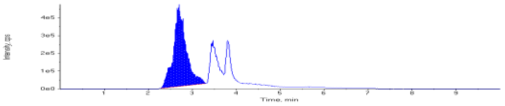	1500.0

Sample 2

Analyte Name	Spectrum	Calculated concentration (ng/ml)
Adenosine	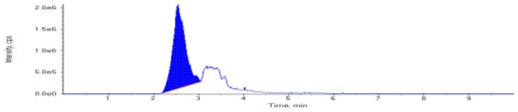	660.00
Cordycepin	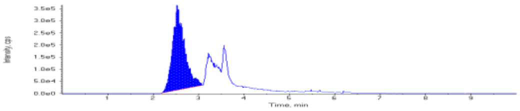	1070.0

Sample 3

Analyte Name	Spectrum	Calculated concentration (ng/ml)
Adenosine	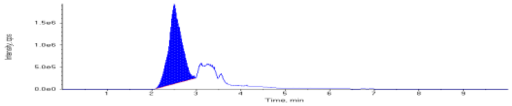	630.00
Cordycepin	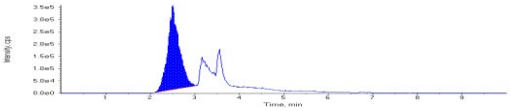	1030.0

Summary (unit : ug/ml)

Calculation Formula: Analyte calculated concentration × 30 (dilution factor) / 1000

Analyte Name	Sample 1	Sample 2	Sample 3
Adenosine	25.65	19.80	18.90
Cordycepin	45.00	32.10	30.90

Sample Spectrum

Sample 1

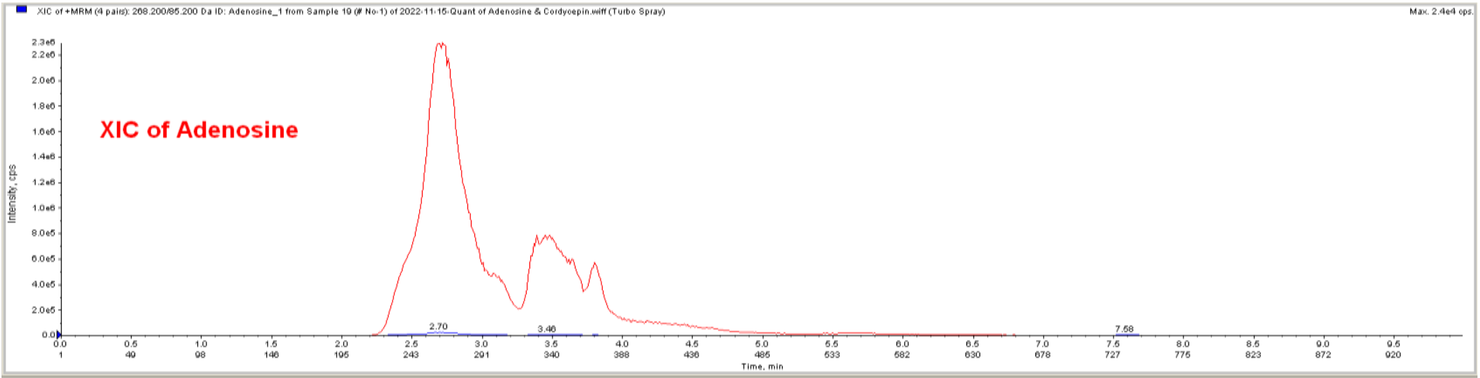

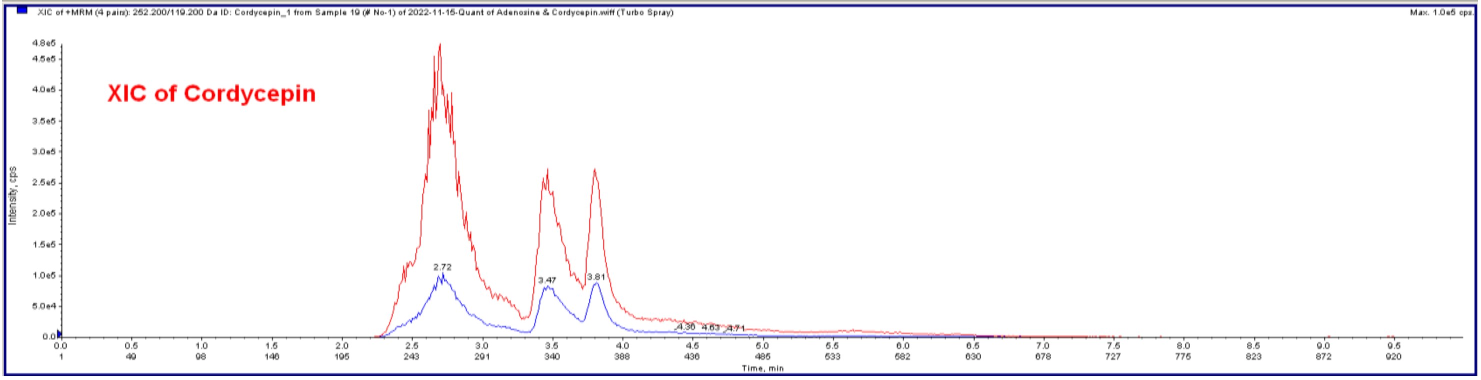

Sample 2

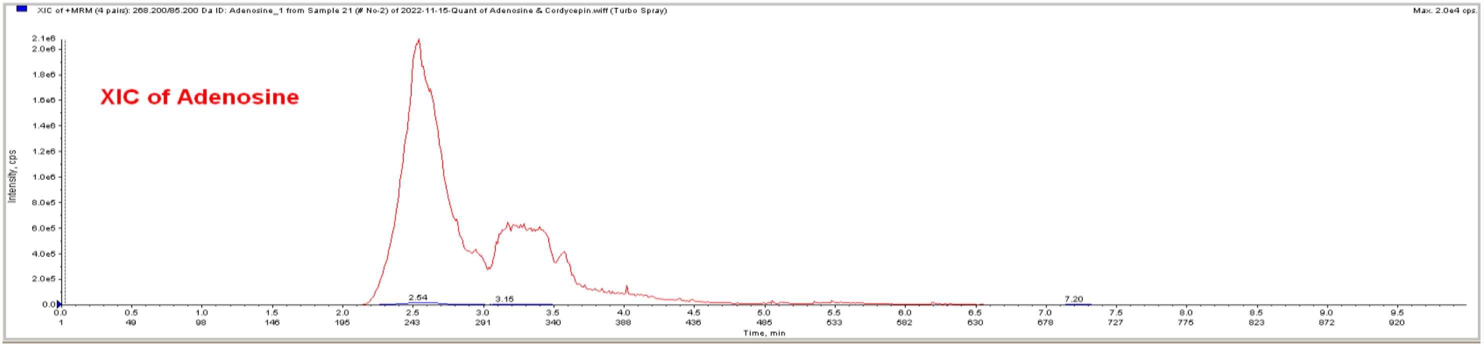

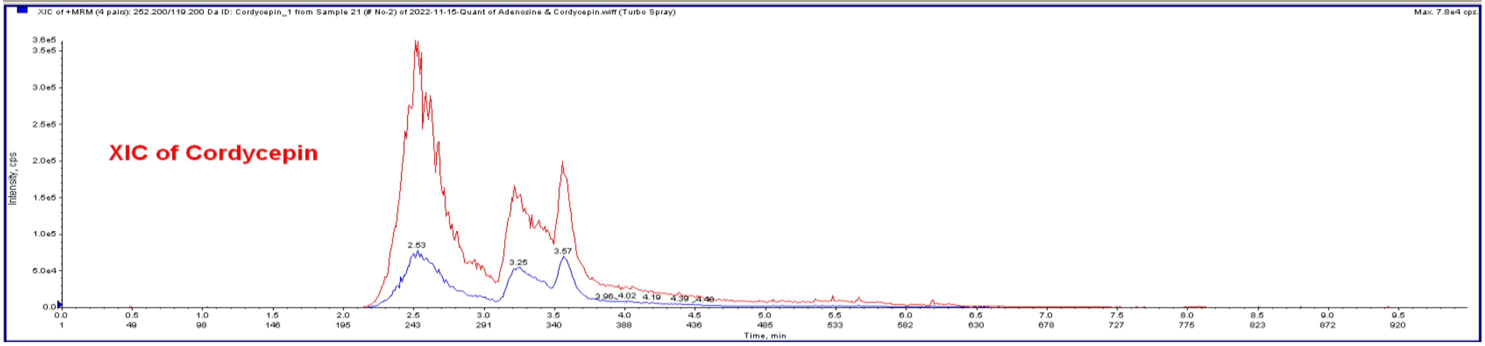

Sample 3

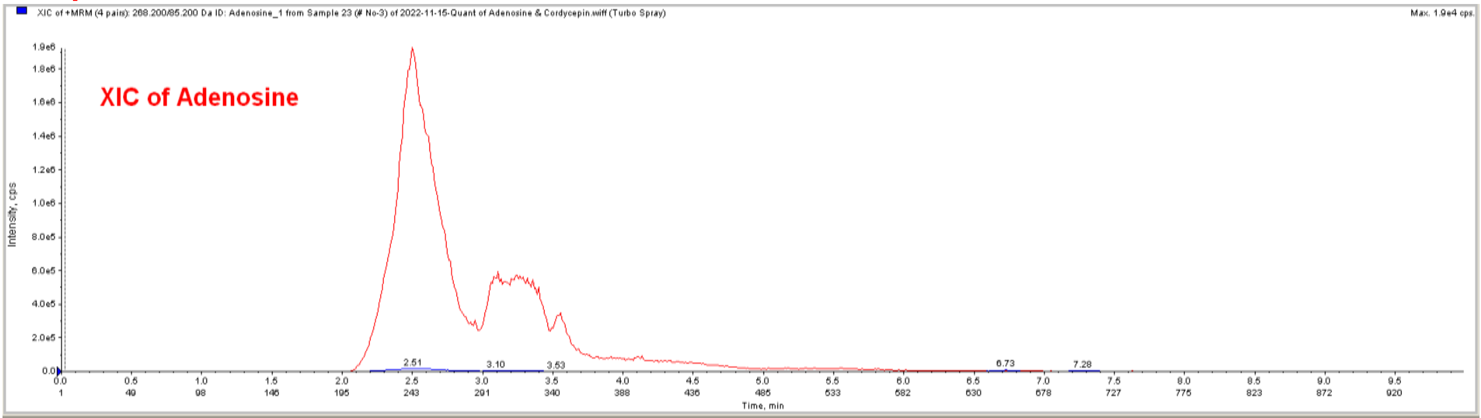
XIC of +MRM (4 pairs): 268.200/85.200 Da ID: Adenosine_1 from Sample 23 (# No-3) of 2022-11-15-Quant of Adenosine & Cordycepin.wiff (Turbo Spray)
Max. 1.9e4 cps.
XIC of Adenosine
Intensity, cps
Time, min

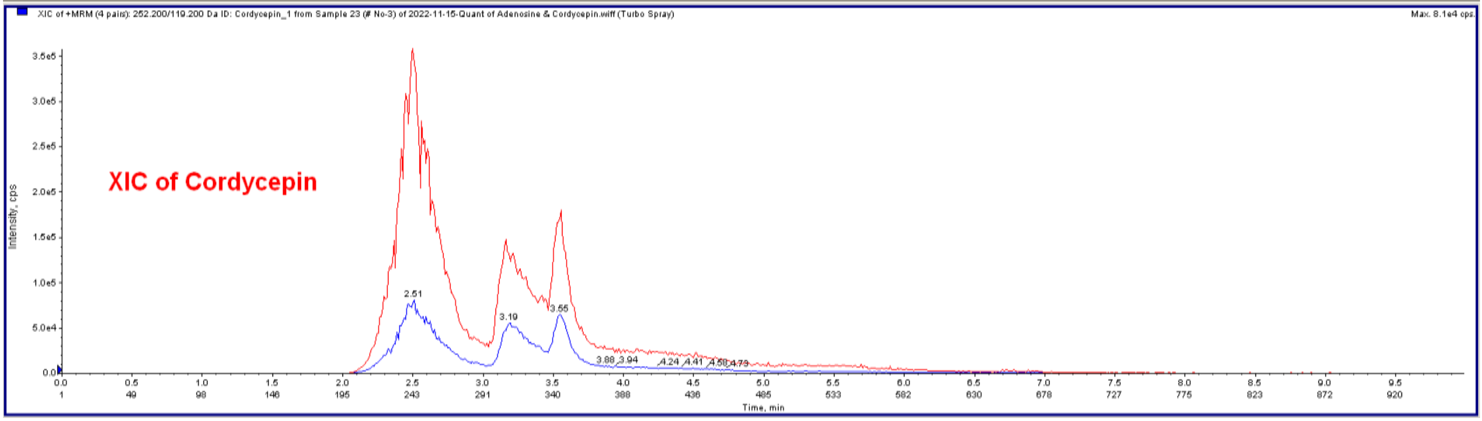
XIC of +MRM (4 pairs): 252.200/119.200 Da ID: Cordycepin_1 from Sample 23 (# No-3) of 2022-11-15-Quant of Adenosine & Cordycepin.wiff (Turbo Spray)
Max. 8.1e4 cps.
XIC of Cordycepin
Intensity, cps
Time, min
